# Ubiquitin-independent degradation of Bim blocks macrophage pyroptosis in sepsis-related tissue injury

**DOI:** 10.1038/s41419-024-07072-z

**Published:** 2024-09-30

**Authors:** Peilin Shi, Yingying Du, Yunyan Zhang, Bo Yang, Qiujing Guan, Yiming Jing, Hao Tang, Jianguo Tang, Chunhui Yang, Xiaoli Ge, Shihui Shen, Lei Li, Chunrong Wu

**Affiliations:** 1 Shanghai Key Laboratory of Regulatory Biology, Institute of Biomedical Sciences, East China Normal University, Shanghai, 200241 China; 2 Department of Respiratory and Critical Care Medicine, Changzheng Hospital, Naval Military Medical University, Shanghai, 200003 China; 3 Joint Center for Translational Medicine, Shanghai Fifth People’s Hospital, Fudan University and School of Life Science, East China Normal University, Shanghai, 200011 China; 4 Department of Trauma-Emergency and Critical Care Medicine Center (TECCMC), Shanghai Fifth People’s Hospital, Fudan University, Shanghai, 200240 China; 5 Department of Emergency, Shanghai Ninth People’s Hospital, Shanghai Jiao Tong University School of Medicine, Shanghai, 200011 China; 6 Department of Emergency Medicine, Xinhua Hospital affiliated to Shanghai Jiao Tong University School of Medicine, Shanghai, 200092 China

**Keywords:** Mechanisms of disease, Cell death and immune response

## Abstract

Pyroptosis, a typical inflammatory cell death mode, has been increasingly demonstrated to have therapeutic value in inflammatory diseases such as sepsis. However, the mechanisms and therapeutic targets of sepsis remain elusive. Here, we reported that REGγ inhibition promoted pyroptosis by regulating members of the gasdermin family in macrophages. Mechanistically, REGγ directly degraded Bim, a factor of the Bcl-2 family that can inhibit the cleavage of GSDMD/E, ultimately preventing the occurrence of pyroptosis. Furthermore, cecal ligation and puncture (CLP)-induced sepsis model mice showed downregulation of REGγ at both the RNA and protein levels. Gasdermin-mediated pyroptosis was augmented in REGγ-knockout mice, and these mice exhibited more severe sepsis-related tissue injury. More importantly, we found that REGγ expression was downregulated in clinical sepsis samples, such as those from patients with *Pseudomonas aeruginosa* (PA) infection. Finally, PA-infected mice showed decreased REGγ levels in the lung. In summary, our study reveals that the REGγ-Bim-GSDMD/E pathway is a novel regulatory mechanism of pyroptosis in sepsis-related tissue injury.

## Introduction

Pyroptosis, a gasdermin-mediated inflammatory programmed death, is critical to immunity. Increasing evidence has suggested its possible role in sepsis [1–4]. It has been reported that gasdermin D (GSDMD) knockout can protect mice against septic shock [5], while a similar relationship has not been reported for other gasdermin family members. Inflammatory caspase-1, murine caspase-11, and human caspase-4/5 can trigger the cleavage of GSDMD and the production of the activated N-terminus to promote membrane pore formation, which is followed by the release of inflammatory cytokines [6–8]. Gasdermin E (GSDME), the most widely studied gasdermin in addition to GSDMD, can be cleaved by caspase-3 and transform TNF- or chemotherapy-induced apoptosis to pyroptosis [9], offering new insights into cancer treatment and antitumor immunity. The Bcl-2 family was found to be the upstream regulatory protein of GSDME. Bax/Bak activate GSDME through caspase-3, while Bcl-2/Mcl-1 inhibits GSDME-mediated pyroptosis [10, 11].

Sepsis, a life-threatening condition in which a dysregulated host response to infection leads to organ dysfunction, is accompanied by high mortality [12, 13]. An analysis conducted by the Global Burden of Disease Study reported more than 40 million sepsis cases and 11 million deaths worldwide in 2017 [14]. The pathogenesis of sepsis is complex, but the mechanisms of sepsis-induced dysfunction have yet to be elucidated [15, 16]. The patient survival rate is low due to the difficulty in early diagnosis and consequent delayed treatment [17–20]. Therefore, new therapeutic targets are urgently needed.

REGγ, also known as PSME3, PA28γ, or Ki antigen, is a member of the proteasomal activators 11S family and promotes protein degradation through a noncanonical proteasome degradation pathway, which functions independently of ATP and ubiquitination by directly binding to the 20S core proteasome [21]. Multiple substrates of REGγ have been discovered, such as cyclin-dependent kinase inhibitor p21 and nuclear receptor cofactor SRC-3 [22, 23]. *REGγ*-knockout cells are more prone to apoptosis, which is manifested by blocking caspase-3 activation and downregulating its substrates [24]. Thus, the REGγ-20S degradation system is involved in many physiological processes, such as the cell cycle, tumor proliferation, migration, angiogenesis, and metastasis [24–29]. Currently, REGγ is gradually becoming a potential cancer biomarker for clinical diagnosis and is used in tumor therapy.

Here, we demonstrate that REGγ regulates gasdermin-mediated pyroptosis by degrading the upstream regulatory protein Bim, a pro-apoptotic protein of the Bcl-2 family, and alleviates sepsis by inhibiting macrophage pyroptosis. In contrast, REGγ knockout accelerates sepsis-induced death, which is accompanied by more severe symptoms. These findings reveal a new regulatory mechanism of sepsis and provide new insights into the treatment of sepsis.

## Results

### REGγ deficiency promoted pyroptotic cell death

**Fig. 1:**
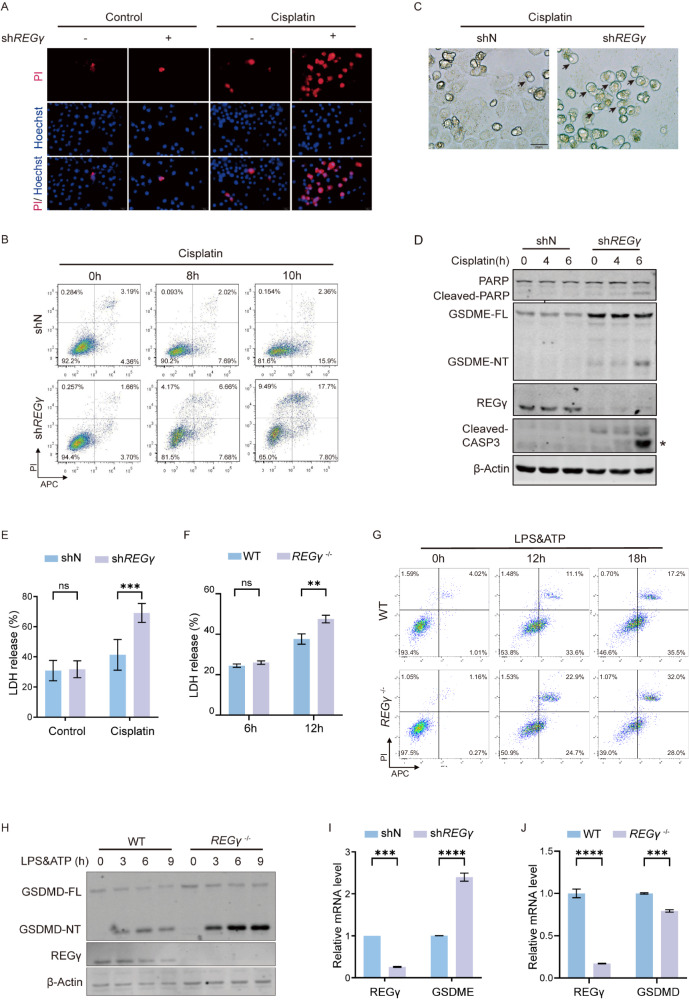
REGγ deficiency promoted pyroptotic cell death. **A** Morphological changes were observed with fluorescence microscopy by Hoechst 33342/PI staining after the 8 h treatment with 40 μM cisplatin in HeLa shN/sh*REGγ* cells (blue fluorescence: Hoechst 33342, red fluorescence: PI). Scale bar, 20 μm. **B** A time-course flow-cytometry analysis of Annexin V–APC and propidium iodide-stained HeLa cells. **C** Cell morphology of HeLa shN cells (left) and sh*REGγ* cells (right) after stimulation with 40 μM cisplatin under the microscope. Scale bar, 20 μm. **D** HeLa cells were treated with 40 μM cisplatin at different times and then analyzed for the expression of the GSDME-mediated pyroptosis pathway by western blot. FL: full length, NT: N-terminus. The asterisk indicates the band being analyzed. **E** LDH release into the HeLa culture medium under cisplatin stimulation was measured. **F** BMDMs were treated with 1 μg/mL LPS priming before 5 mM ATP for 6 h and 12 h. LDH release into the cell culture medium was measured. Data are means ± SD taken from three technical replicates. **G** Flow cytometry of Annexin V-APC and propidium iodide-stained BMDMs. **H** BMDMs were pre-priming with 1 μg/mL LPS at different time points before being stimulated with 5 mM ATP. GSDMD-mediated pyroptosis pathways were analyzed by western blot. **I** mRNA levels of GSDME were assessed by qPCR in HeLa cells. **J** mRNA levels of GSDMD were assessed by qPCR in BMDMs. Differences were measured by two-way ANOVA. Data are means ± SD taken from three technical replicates. ns, not significant, *P < 0.05, **P < 0.01, ***P < 0.001, ****P < 0.001.

Several studies have reported a correlation between decreased REGγ levels and elevated apoptosis [24, 30, 31]. Research has indicated that *REGγ*-knockout (*REGγ*^-/-^) mouse embryonic fibroblasts show markedly increased apoptosis [32]. We first generated HeLa cell lines stably expressing a control shRNA (shN) or a *REGγ*-specific knockdown shRNA (sh*REGγ*) (Fig. S1A). Propidium iodide (PI) and Hoechst 33342 staining were performed to distinguish apoptotic cells with impaired membrane integrity from viable cells. Following a 6-h exposure to cisplatin, HeLa sh*REGγ* cells displayed significantly higher PI absorption than HeLa shN cells, suggesting a greater tendency for cell death in REGγ-null cells, accompanied by membrane rupture (Fig. 1A and Fig. S1B). In addition, a time-course study using Annexin V-APC and PI staining in flow-cytometry analysis revealed a similar conclusion. The proportion of Annexin V and PI double-positive dead cells significantly differed in HeLa shN and sh*REGγ* cells, with the latter being higher (Fig. 1B). Interestingly, HeLa sh*REGγ* cells tended to exhibit positive double-staining rather than typical Annexin V-positive staining, which indicates early apoptosis. Thus, we observed the morphology of HeLa cells treated with cisplatin. Under the same conditions, both HeLa shN and sh*REGγ* cells showed large bubbles caused by plasma membrane swelling, which is a typical characteristic of pyroptosis. Notably, HeLa sh*REGγ* cells showed a higher prevalence of pyroptosis-like cell morphology than HeLa shN cells (Fig. 1C). Subsequently, we recorded movies to investigate the dynamics of cell death, and we observed that cells swelled until they ruptured, with this phenomenon being particularly evident in HeLa sh*REGγ* cells (Movies S1 and S2). These findings prompted us to consider whether the absence of REGγ accelerates pyroptosis.

Pyroptosis is an inflammatory programmed cell death mediated by the gasdermin family, of which GSDMD and GSDME are widely studied [33]. The protein expression of GSDMD and GSDME varies among different cell types. Our findings revealed high expression of GSDME in Hela, HaCat, and HK-2 cells, while GSDMD expression was elevated in BMDM, MH-S, and PM cells. (Fig. S1C). As reported, GSDME was found to facilitate pyroptosis in HeLa cells induced by chemotherapy drugs, whereas GSDMD-mediated pyroptosis in BMDMs induced by the combination of LPS (lipopolysaccharide) and ATP [6, 9, 34]. We evaluated GSDMD/E cleavage in HeLa shN and sh*REGγ* cells following successful pyroptosis induction with cisplatin. The result demonstrated the upregulation of GSDME cleavage in HeLa sh*REGγ* cells (Fig. S1D). The dominance of cisplatin-induced pyroptosis by GSDME, rather than GSDMD, is likely due to the significantly lower expression of GSDMD in HeLa cells compared to GSDME (Fig. S1C). As expected, GSDME was cleaved within 12 h, both in the presence and absence of REGγ, triggering pyroptosis. Notably, this cleavage occurred within a much shorter timeframe in HeLa sh*REGγ* cells (Fig. S1E). This finding suggested that the absence of REGγ can accelerate the process of pyroptosis. In HeLa sh*REGγ* cells, both the full-length GSDME (GSDME-FL) and its N-terminal cleavage product (GSDME-NT) were upregulated upon cisplatin stimulation. Caspase-3 was activated, and PARP was cleaved, consistent with a previous study [35] (Fig. 1D). REGγ also significantly decreased the release of lactate dehydrogenase (LDH), indicating that the inhibition of REGγ worsened cell membrane integrity interruption, further confirming the higher percentage of lytic cell death (Fig. 1E). These observations demonstrated that REGγ deficiency enhanced chemotherapy drug-induced pyroptosis and sped up the process.

Additionally, we validated the regulatory role of REGγ on pyroptosis in other GSDME-positive cell lines, including HaCat and HK-2 cells (Fig. S1C). More intense cleaved GSDME was observed in HaCat sh*REGγ* cells, accompanied by more LDH release (Fig. S1F, G). The same conclusion was validated in HK-2 cells, where the silencing of *REGγ* led to increased expression of GSDME-NT (Fig. S1H). Both HaCat cells and HK-2 cells showed slight GSDMD cleavage because of the much lower expression of GSDMD, like HeLa cells. These results verified that REGγ inhibited GSDME-mediated pyroptosis.

Similar findings were observed in primary mouse bone marrow-derived macrophages (BMDMs). We used conventional methods to induce GSDMD-mediated pyroptosis in macrophages [36]. Briefly, BMDMs from wild-type (WT) mice and *REGγ*^-/-^ mice were treated with LPS priming before ATP administration. Afterward, we measured LDH release in the BMDMs culture medium. There was a significant difference at 12 h between WT and *REGγ*^-/-^ BMDMs, with the latter exhibiting higher levels of LDH (Fig. 1F). Consistent with this result, flow-cytometry analysis showed a higher percentage of Annexin V and PI double-stained cells in *REGγ*^*-/-*^ BMDMs (Fig. 1G). Moreover, western blot analysis indicated the cleavage of GSDMD in BMDMs after combined treatment with LPS and ATP. *REGγ*^-/-^ BMDMs exhibited upregulated expression of GSDMD-NT, indicating more severe pyroptotic cell death (Fig. 1H). We also validated the conclusion that REGγ regulates GSDMD-mediated pyroptosis in other macrophages. Primary peritoneal macrophages (PMs) from *REGγ*^-/-^ mice accelerated the cleavage of GSDMD, compared with *REGγ-*WT PMs, indicating more intense GSDMD-mediated pyroptosis (Fig. S1I). Taken together, our results demonstrate that REGγ regulates GSDMD-mediated pyroptosis in macrophages. Considering the role of REGγ in protein degradation, we measured the mRNA level of GSDME to preliminarily ascertain the potential for REGγ-mediated degradation of GSDME. However, the upregulation of GSDME after *REGγ* knockdown was, in fact, a result of heightened transcriptional activity, as evidenced by the elevated mRNA levels (Fig. 1I). GSDMD also differed at mRNA level between WT BMDMs and *REGγ*^-/-^ BMDMs (Fig. 1J). These results indicated that neither GSDME nor GSDMD is the degradation substrate of REGγ, suggesting that there was another unidentified direct substrate for the regulation of pyroptosis by REGγ.

Overall, these observations demonstrated that REGγ inhibition promoted pyroptosis through the indirect regulation of GSDME and GSDMD in both macrophages and tumor cells.

### Loss of Bim alleviated the promotive effect of REGγ deficiency on pyroptosis

**Fig. 2:**
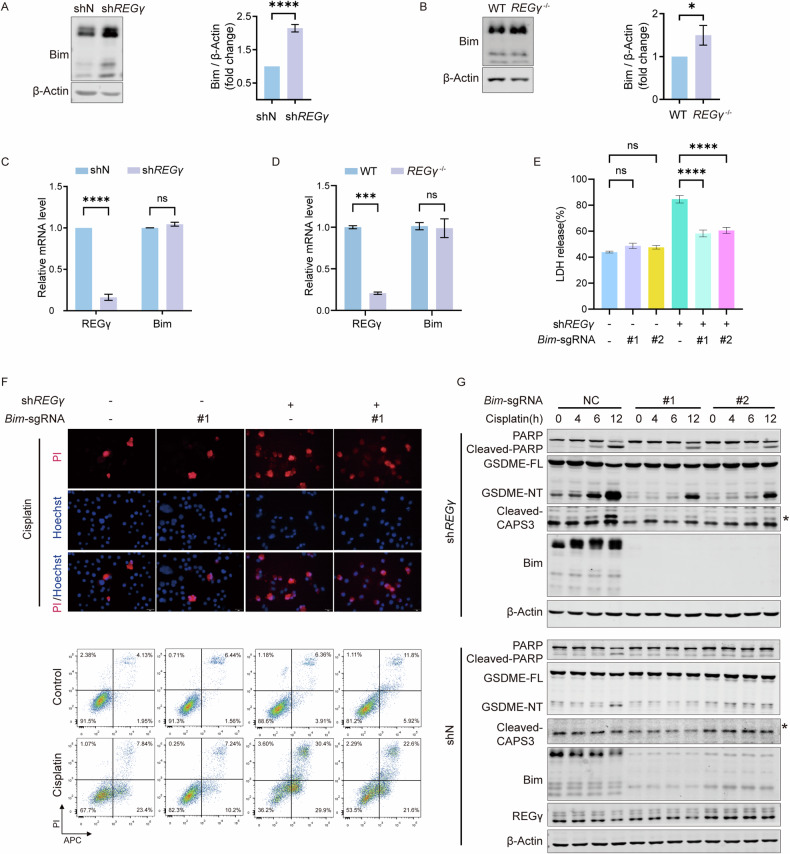
Loss of Bim alleviated the promotive effect of REGγ deficiency on pyroptosis. **A** Bim expression in HeLa was assessed by western blot and semiquantitative analysis. Differences were measured by *t*-test. **B** Bim expression in BMDMs was assessed by western blot and semiquantitative analysis. Differences were measured by *t-*test. **C** mRNA level of Bim in HeLa cells was analyzed by qPCR. Differences were measured by two-way ANOVA. **D** mRNA level of Bim in BMDMs was analyzed by qPCR. Differences were measured by two-way ANOVA. **E** LDH release was measured in HeLa cells treated with 40 μM cisplatin for 14 h. #1/2 means cells were transfected with *Bim*-specific sgRNAs. Differences were measured by one-way ANOVA. **F** Cell morphology of HeLa cells after stimulation with 40 μM cisplatin under a microscope. Scale bar, 20 μm (up). Cell death mode was shown with flow cytometry of Annexin V-APC and propidium iodide-stained HeLa cells (down). **G** HeLa cells were treated with 40 μM cisplatin and then analyzed by western blot. NC means cells were transfected with sgRNA negative control. #1/2 means cells were transfected with *a Bim*-specific sgRNAs. The asterisk indicates the band being analyzed. Data are means ± SD taken from three technical replicates. ns, not significant, *P < 0.05, **P < 0.01, ***P < 0.001, ****P < 0.001.

To explore the underlying mechanism of elevated pyroptosis, we investigated a family of proteins that is related to cell death and upstream of GSDME, the Bcl-2 family. It has been reported that the Bcl-2 family can regulate GSDME through the activation of caspase-3, revealing its role in the regulation of pyroptosis [11]. Members of the Bcl-2 family can be divided into pro-apoptotic, pro-survival, and execution proteins [37]. In view of the inhibitory effect of REGγ on pyroptosis, we speculated that REGγ might execute its effects by regulating the Bcl-2 family through degradation. Therefore, the expression levels of those proteins in the Bcl-2 family were measured in REGγ-deficient HeLa cells and BMDMs (Fig. S2A–D). The protein levels of pro-apoptotic proteins were upregulated caused by the inhibition of REGγ, but the changes in mRNA levels were different from each other. As we know, REGγ regulates substrates only at the protein level, we focused on a pro-apoptotic protein, Bim, which can activate the homo-oligomerization of Bax/Bak to promote cell death [38]. Bim was found to be significantly upregulated, without accompanying changes in the RNA levels in HeLa cells and BMDMs (Fig. 2A–D). However, the role of Bim in pyroptotic cell death has yet to be reported.

To prove whether Bim plays a role in pyroptosis regulation, *Bim* sgRNAs were designed, and HeLa *Bim*-knockout cells and HeLa *Bim*-knockout-sh*REGγ* cells were obtained through CRISPR-Cas9 technology (Fig. S2E). The knockout of Bim alleviated cell membrane integrity interruption caused by the knockdown of REGγ in HeLa cells (Fig. 2E). Similarly, compared to HeLa sh*REGγ* cells, HeLa *Bim*-knockout-sh*REGγ* cells exhibited significantly reduced pyroptotic characteristics, as demonstrated by Hoechst 33342/PI staining and flow-cytometry analysis (Fig. 2F and Fig. S2F). Consistent with previous findings, GSDME-NT was detected in HeLa sh*REGγ* cells within 6 h after cisplatin treatment, while there was almost no GSDME-NT in HeLa shN cells within 8 h. In HeLa *Bim*-knockout-sh*REGγ* cells, GSDME-NT production continued under cisplatin stimulation but was significantly reduced and delayed, coinciding with a decrease in activated caspase-3, suggesting a reduced degree of pyroptosis (Fig. 2G). Silencing Bim partially inhibited cisplatin-induced pyroptosis in HeLa cells. As the upstream regulatory protein of the mitochondrial apoptotic pathway, Bim knockout also affected apoptosis by inhibiting the expression of cleaved PARP (Fig. 2G).

In order to define the in vivo function of Bim, we generated the *Bim* conditional knockout mice (*Bim*^flox/flox^) (Fig. S2G). Next, we extracted BMDMs from *Bim*^flox/flox^*LysM*^Cre^ mice (*Bim*^LysM^ BMDMs) generated by crossing *Bim*^flox/flox^*LysM*^+/+^ mice with *LysM*-Cre mice. Comparing with BMDMs from *Bim*^flox/flox^*LysM*^+/+^ mice (*Bim-*WT BMDMs), the deficiency of Bim decreased the cleavage of GSDMD in *Bim*^LysM^ BMDMs, as well as decreased the activation of caspase-1 (Fig. S2H).

Collectively, these findings demonstrated that downregulating Bim expression can alleviate the increased pyroptosis induced by REGγ deficiency.

### REGγ mediated ubiquitin-ATP-independent degradation of Bim

**Fig. 3:**
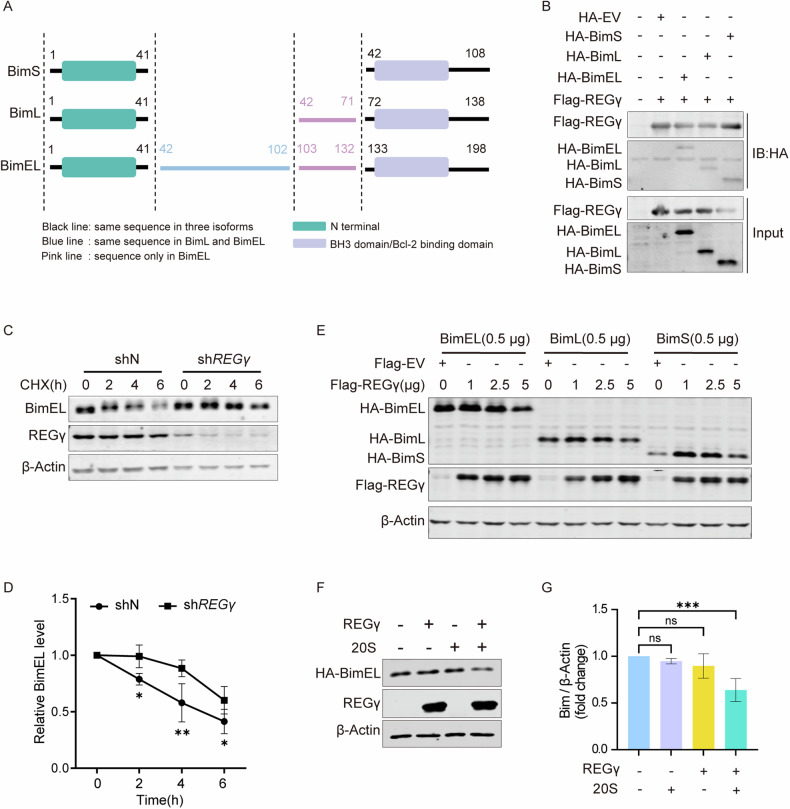
REGγ mediated ubiquitin-ATP-independent degradation of Bim. **A** Schematic diagram of amino acid sequence for three main isoforms of Bim, including BimEL, BimL, and BimS. **B** The physical interactions between REGγ and Bim were defined by reciprocal co-immunoprecipitation followed by western blot analysis. 293 T cells were transiently expressed 4 μg of Flag-REGγ, 2 μg of HA- BimEL (and BimL, BimS), or 2 μg of the control HA-vector. **C** HeLa shN cells and HeLa sh*REGγ* cells were treated with 100 μg/mL cycloheximide (CHX) for the indicated times followed by western blot analysis. **D** The degradation rate of Bim is visualized by a statistical line graph. Differences were measured by two-way ANOVA. **E** Flag-REGγ was transfected into 293T KO (*REGγ* knockout) cells, accompanied by HA-Bim. The expression of Bim plasmids was assessed by western blot. **F** Bim was translated in vitro in TNT® Quick Coupled Transcription/Translation System. And then incubated for 4 h at 37 °C with purified protein REGγ and 20S proteasome. The degradation of Bim was assessed by western blot. **G** Quantitated results in **F** show Bim degradation changes. Differences were measured by ordinary one-way ANOVA. Data are means ± SD taken from three technical replicates. ns, not significant, *P < 0.05, **P < 0.01, ***P < 0.001.

We have demonstrated the role of Bim in pyroptosis, but how REGγ regulates Bim is still uncertain. Bim can be degraded independently of ubiquitination [39]. Considering the ability of REGγ to degrade intact proteins, further investigations were conducted to determine whether Bim is a novel target of REGγ-mediated proteasome degradation. As mentioned above, REGγ can affect the protein levels of Bim without disrupting mRNA transcription (Fig. 2A). Analysis of Bim expression in the colon, cerebellum, and liver revealed significant differences between WT and *REGγ*^-/-^ mice, providing evidence for the protein-modulating role of REGγ on Bim (Fig. S3A–C). Bim has three main isoforms, BimS, BimL, and BimEL, which have relatively high sequence similarity at N-terminal and C-terminal (Fig. 3A and Fig. S3D). Physical interaction between REGγ and Bim has been demonstrated in reciprocal co-immunoprecipitation assays, with REGγ binding to all the three major isoforms of Bim (Fig. 3B). Bim was significantly stabilized in the absence of REGγ under the treatment of protein synthesis inhibitor cycloheximide (CHX). Inhibition of REGγ significantly decelerated the degradation of Bim, confirming the role of REGγ in promoting Bim degradation (Fig. 3C, D). Different concentrations of REGγ plasmids were transfected into REGγ-null expression 293T cells, accompanied by a certain concentration of Bim plasmids, and the results indicated that the expression of Bim was negatively correlated with the concentration of REGγ (Fig. 3E). Furthermore, REGγ was purified to degrade Bim in vitro. In vitro translated Bim was incubated with purified REGγ and 20S proteasome, showing a direct degradation of Bim by the REGγ-20S proteasome (Fig. 3F, G).

Altogether, these findings collectively demonstrated that Bim is a substrate of REGγ that undergoes degradation by the REGγ-20S proteasome in a ubiquitination- and ATP-independent manner.

### Cecal ligation and puncture (CLP)-induced sepsis downregulated the expression of REGγ

**Fig. 4:**
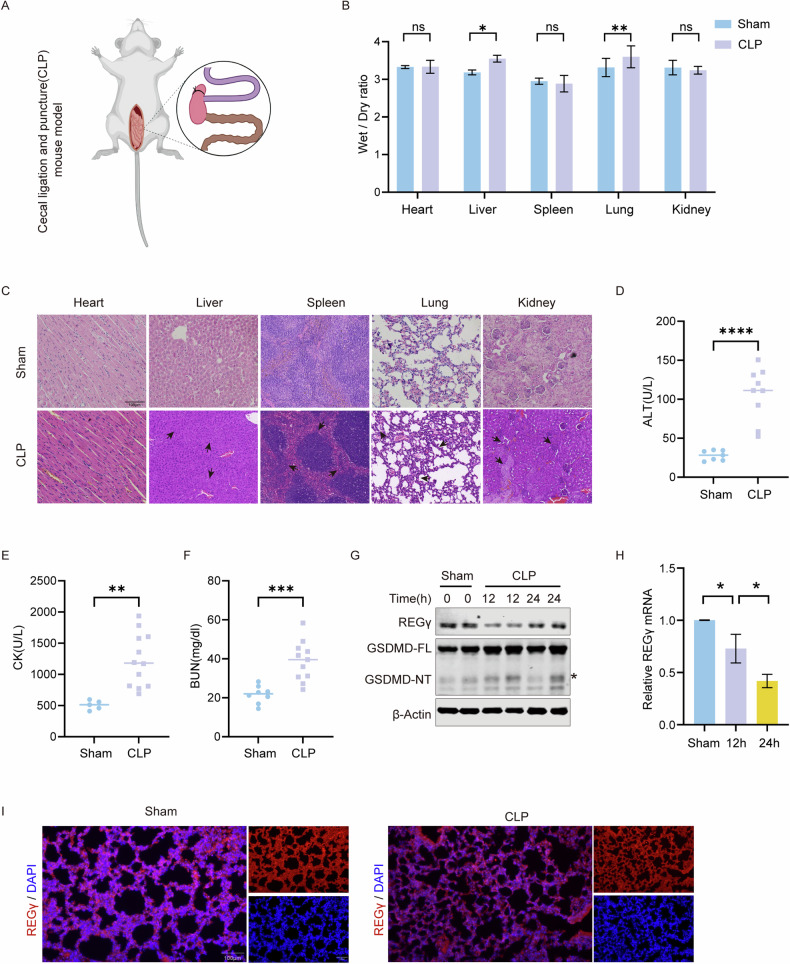
Cecal ligation and puncture (CLP)-induced sepsis downregulated the expression of REGγ. **A** Schematic diagram of mice CLP-induced sepsis model. **B** The wet-dry ratio of major organs from each group. Differences were measured by two-way ANOVA. **C** H&E staining of liver, kidney, lung, heart, and spleen tissue from 6-10 weeks C57BL/6 male wild-type mice and CLP mice. Black arrows indicate the organ-damaged areas. Scale bar, 100 μm. **D**–**F** Blood serum ALT, CK, and BUN levels from each group, respectively. Each spot represents a sample from an individual mouse. Differences were measured by *the t*-test. **G** Mice were sacrificed after 12 or 24 h. BMDMs were extracted from the sham group and CLP group for western blot analysis. Short stands for short exposure and long stands for long exposure. The asterisk indicates the band being analyzed. **H** mRNA level of REGγ was assessed by qPCR in BMDMs. Differences were measured by ordinary one-way ANOVA. **I** Immunofluorescence of REGγ in the lung in CLP-induced WT sepsis mice. Scale bar, 100 μm. Data are means ± SD taken from three technical replicates. ns, not significant, *P < 0.05, **P < 0.01, ***P < 0.001, **** *P* < 0.0001.

Increasing evidence supports the relationship between pyroptosis and sepsis [2, 40, 41]. Interestingly, the absence of REGγ in hematopoietic cells increases the likelihood of bacterial infection in the host [42]. This finding led us to question whether the regulation of REGγ in pyroptosis might influence host infectious diseases such as sepsis. To investigate the role of REGγ in sepsis, an in vivo sepsis model was established in 6- to 10-week-old C57BL/6 WT male mice through CLP [43] (Fig. 4A). Mice were divided into the sham operation group and the CLP group. By comparing the wet-dry weight ratios of the two groups, it was found that edema was present in the liver and lung of CLP-induced sepsis mice (Fig. 4B). Tissue injuries were intuitively observed via hematoxylin and eosin (H&E) staining. The data indicated edema in the liver and kidney, widespread and reduced white pulp in the spleen, thickening of the alveolar wall in the lungs with the formation of a transparent membrane, and inflammatory cell infiltration in various tissues (Fig. 4C). Serum was extracted from mouse whole blood to analyze markers indicating organ dysfunction. Alanine aminotransferase (ALT), creatine kinase (CK) and blood urea nitrogen (BUN) levels indicate damage to the liver, heart, and kidney, respectively. These indicators were elevated in CLP-induced sepsis model mice, suggesting that multiple organ damage consistent with sepsis symptoms was occurring (Fig. 4D–F). These results suggest that sepsis was successfully induced by CLP.

Thereafter, we observed the expression of REGγ following CLP operation. Western blot analysis revealed a remarkable downregulation of REGγ. Concurrently, we found an increase in GSDMD-NT expression levels, indicating the presence of pyroptosis in these samples with downregulated REGγ expression (Fig. 4G). Additionally, we examined the transcript levels of REGγ and found a significant reduction in the CLP group (Fig. 4H). Immunofluorescence in the lung also showed that the reduced expression of REGγ in CLP-induced sepsis mice (Fig. 4I). Furthermore, we investigated transcription factors to identify the reasons for the transcriptional downregulation of REGγ. We identified some reported transcription factors that regulate the transcription of REGγ [42, 44, 45] and found that *Fos* was significantly downregulated (Fig. S4).

Gasdermin-mediated pyroptosis plays a crucial role in various conditions, including sepsis, as previously mentioned, as well as toxic adverse effects caused by chemotherapy. *GSDME*^*-/-*^ mice exhibited milder immune dysfunction following cisplatin injections than WT mice [9]. The inhibitory effect of REGγ on pyroptotic cell death was further explored, leading to the investigation of the role of REGγ in the toxicity of chemotherapy drugs. The small intestine, lung, and kidney of both *REGγ*^*-/-*^ mice and WT mice were collected following peritoneal injection of cisplatin. H&E staining revealed significant disruptions in both crypts and villi of the injected mice, accompanied by noticeable immune cell infiltration. Similarly, lung and kidney injuries were observed. In *REGγ*^*-/-*^ mice, the severity of these organ injuries was heightened, underscoring that REGγ could partially mitigate the toxicity of chemotherapy drugs (Fig. S5A). This effect could be caused by an increase in lymphocytes in the spleen, which could enhance pyroptosis (Fig. S5B). However, REGγ deficiency did not impact body weight (Fig. S5C). We also detected a decrease in REGγ protein levels in lung tissues from WT mice with cisplatin treatment, but not in the kidney (Fig. S5D, E). Overall, we demonstrated the ability of REGγ to mitigate the toxicity induced by chemotherapy drugs. This effect might stem from the inhibitory impact of REGγ on pyroptosis.

These data demonstrated a negative correlation between REGγ and pyroptosis and thereby sepsis, suggesting that REGγ plays an important role in the development of sepsis by regulating pyroptosis.

### REGγ expression decreased in clinical samples of patients with sepsis

**Fig. 5:**
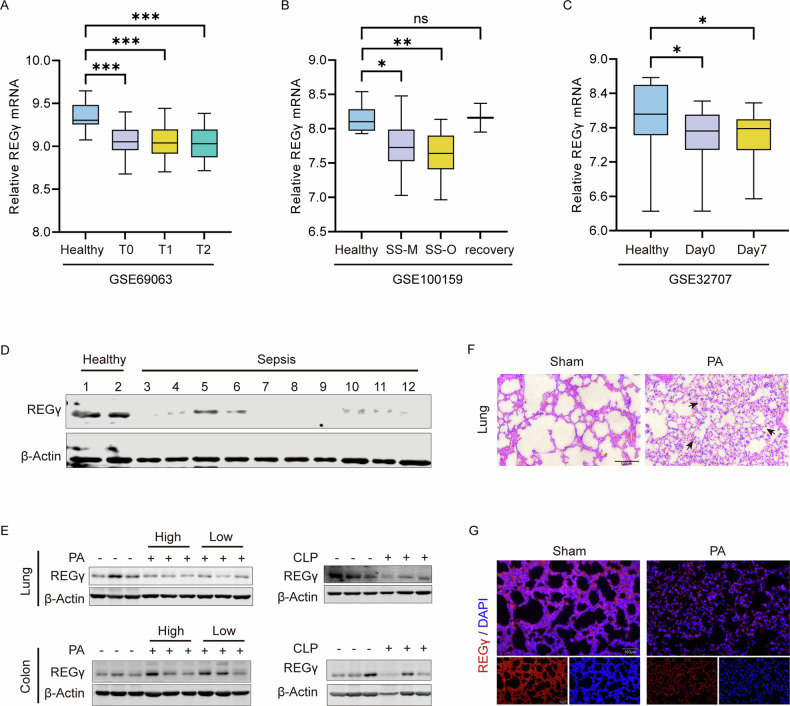
REGγ expression decreased in clinical samples of patients with sepsis. **A**, **B** The mRNA level of REGγ in the peripheral blood samples from healthy controls (Healthy) and sepsis patients (SS). **A** T0 represents the blood samples collected immediately after receiving patients, T1 represents the blood samples collected 1 h after receiving patients, and T2 represents the blood samples collected 3 h after receiving patients. Data was derived from the GEO database GSE69063. **B** SS-M represents blood samples from patients with melioidosis infection and SS-O represents blood samples from patients with other infections. Recovery represents blood samples from recovered sepsis patients. The data was derived from the GEO database GSE100159. **C** Day0 represents the blood samples collected on the first day after receiving patients, and Day7 represents the blood samples collected on the seventh day after receiving patients. The data was derived from the GEO database GSE32707. Differences were measured by ordinary one-way ANOVA. **D** Human peripheral blood mononuclear cells (PBMCs) were extracted from blood samples of patients with sepsis, which were then subjected to western blot. **E** *Pseudomonas aeruginosa* (PA) was injected intraperitoneally into wild-type mice. Lung and colon tissue were extracted from each group for western blot analysis (left). High means higher PA injected concentration 1 × 10^6^ CFU/mL, and low means lower PA injected concentration 2 × 10^5^ CFU/mL. Lung and colon tissue from CLP WT mice were extracted from each group for western blot analysis (right). **F** H&E staining of lung tissues from 6-10 weeks C57BL/6 male wild-type mice with PA infection. Black arrows indicate the organ-damaged areas. Scale bar, 100 μm. **G** Immunofluorescence of REGγ in the lung tissue in PA-infected mice. Scale bar, 100 μm. Data are means ± SD taken from three technical replicates. ns, not significant, *P < 0.05, **P < 0.01, ***P < 0.001, **** *P* < 0.0001.

Excessive inflammation induced by pyroptosis is one of the leading causes of death in patients with sepsis [46]. Considering the regulatory effect of REGγ on pyroptosis, we obtained RNAseq analysis data on sepsis patient blood samples from the GEO database and conducted a comparative analysis of REGγ expression. The results showed a significant decrease in REGγ expression levels in the whole blood samples of sepsis patients compared to healthy individuals (Fig. 5A–C). Two patients who recovered from sepsis in the GSE100159 database showed restored REGγ expression, suggesting a regulatory role of REGγ in sepsis (Fig. 5B). We then collected clinical blood samples from sepsis patients and healthy volunteers without underlying disease as a control. Human peripheral blood mononuclear cells (PBMCs) were isolated. Analysis revealed that REGγ expression was downregulated in clinical sepsis samples, suggesting that REGγ indeed affects patients with sepsis under certain causative factors (Fig. 5D). Patient information showed that sepsis patients were mostly infected with bacteria causing lung injury. We chose *Pseudomonas aeruginosa* (PA) to independently infect WT mice with two different concentrations and examine the expression of REGγ (Fig. 5E). A downregulated expression of REGγ was found in the lung but not in the colon. Similarly, the expression of REGγ in the lung, colon, and small intestine after the CLP operation was assessed by western blot analysis. REGγ was only significantly downregulated in the lung (Fig. 5E and Fig. S6). H&E staining showed a severe lung injury and the result of immunofluorescence in lung tissue also showed a reduction of REGγ in PA infection mice (Fig. 5F, G). In summary, REGγ is downregulated in sepsis and may influence lung damage caused by sepsis.

### CLP-induced sepsis is exacerbated by the null expression of REGγ

**Fig. 6:**
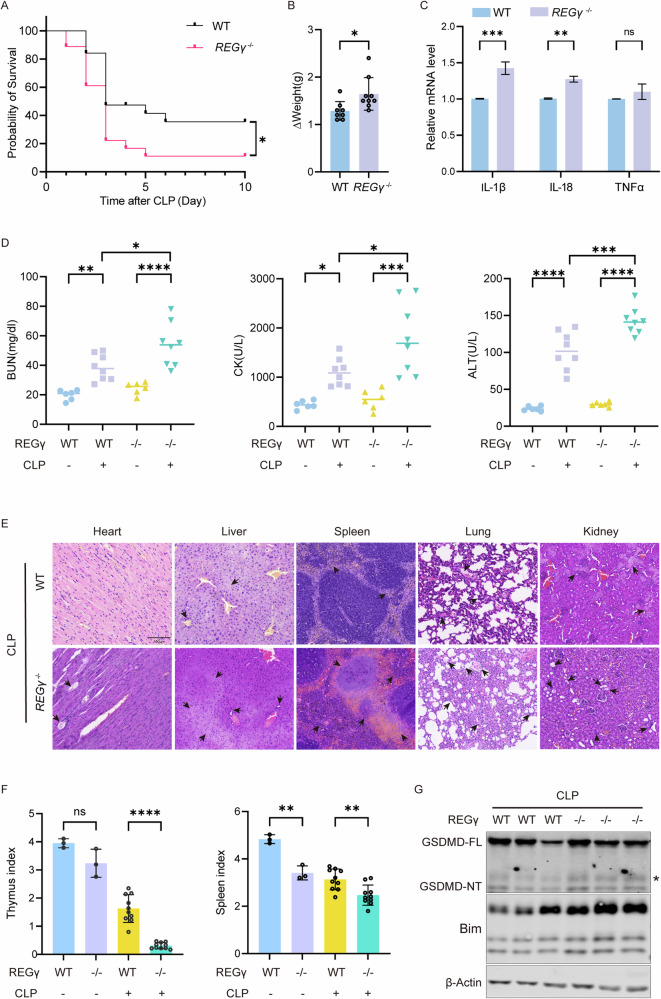
CLP-induced sepsis is exacerbated by the null expression of REGγ. **A** Survival curves of CLP-induced sepsis WT/ *REGγ*^-/-^ mice within 10 days compared with the sham group, *n* = 18. Differences were measured by the Log-rank test. **B** Mouse weight when sacrificed is shown relative to weight before CLP operation. Each spot represents a sample from an individual mouse. Differences were measured by the *t*-test. **C** mRNA level of IL-1β, IL-18, and TNFα of primary BMDMs from wide-type mice and *REGγ*^*-/-*^ mice with CLP-induced sepsis. Differences were measured by two-way ANOVA. **D** Blood serum BUN, CK, and ALT levels from each group, respectively. Each spot represents a sample from an individual mouse. Differences were measured by two-way ANOVA. **E** H&E staining of heart, liver, spleen, lung, and kidney tissue from 6–10 weeks C57BL/6 male wild-type mice and CLP mice. Black arrows indicate the organ-damaged areas. Scale bar, 100 μm. **F** Statistics for thymus weights relative to the weight of mice from each group. Differences were measured by two-way ANOVA. **G** Western blot analysis of BMDMs from each group. The asterisk indicates the band being analyzed. Data are means ± SD taken from three technical replicates. ns, not significant, *P < 0.05, **P < 0.01, ***P < 0.001, ****P < 0.0001.

As a key protein in the nonclassical proteasome degradation pathway, REGγ has been gradually recognized for its therapeutic value in various diseases [44, 47, 48]. To further define the potential therapeutic value of REGγ in sepsis, we constructed a CLP sepsis model using laboratory-inherent *REGγ*^-/-^ C57BL/6 mice and WT C57BL/6 mice as controls. Within 10 days after the CLP operation, a significantly higher mortality rate was observed in the *REGγ*^-/-^ mice than in the WT mice (Fig. 6A). Comparing the weight of mice before and after the CLP operation, we found that *REGγ*^-/-^ mice lost less weight than WT mice (Fig. 6B). Subsequently, we measured the mRNA levels of IL-1β, IL-18 and TNFα in *REGγ*^-/-^ BMDMs and WT BMDMs isolated from CLP mice. Compared to WT mice, *REGγ*^-/-^ mice produced more inflammatory factors after the CLP operation, suggesting a severe inflammatory reaction (Fig. 6C). The blood of sepsis mice was collected for biochemical analysis. Significantly upregulated BUN, CK, and ALT levels were observed in both *REGγ*^-/-^ mice and WT mice, indicating kidney, heart, and liver injury, respectively, with higher levels in *REGγ*^-/-^ mic (Fig. 6D). H&E staining revealed more severe tissue damage in *REGγ*^-/-^ sepsis mice, including severer numerous cell deaths and inflammatory cell infiltration (Fig. 6E). These data all demonstrated that the elimination of REGγ leads to more severe sepsis-related symptoms, including inflammation and tissue damage, highlighting the crucial role of REGγ in sepsis development.

The indices of spleen and thymus can reflect the integrity of the immune system. We compared the spleen index and thymus index between the sham group and the CLP group (Fig. 6F). The immune system of both groups was weakened according to the declining spleen index and thymus indices. The much lower indices in *REGγ*^-/-^ mice indicated that REGγ also affected the immune function of mice. Consistent with the above conclusion, we observed increased macrophage pyroptosis in *REGγ*^-/-^ sepsis mice, as evidenced by the higher expression of GSDMD-NT in *REGγ*^-/-^ BMDMs. Additionally, Bim, the upstream regulator of pyroptosis studied in this paper, was also highly expressed in *REGγ*^-/-^ BMDMs (Fig. 6G).

These observations provide evidence that the modulatory effect of REGγ on pyroptosis can influence the severity of sepsis, offering new insights into its treatment.

## Discussion

The storm of inflammatory factors caused by excessive pyroptosis is a common cause of death in sepsis [46, 49]. The present study showed that REGγ exerted inhibitory effects on pyroptosis. When inducing GSDME-mediated pyroptosis in HeLa cells with cisplatin, HeLa cells lacking REGγ underwent pyroptosis more rapidly and exhibited a higher propensity toward pyroptosis. Similarly, in the LPS-ATP-induced GSDMD-mediated pyroptosis model, loss of REGγ led to enhanced pyroptotic death of BMDMs, which was manifested by the heightened release of cellular contents and upregulated expression of inflammatory factors. These findings indicate that REGγ indeed plays a discernible inhibitory role in the induction of pyroptotic cell death.

REGγ, a proteasome activator, was able to degrade intact proteins independent of ubiquitination and ATP. This discovery introduced a novel dimension into the realm of protein degradation research. REGγ exhibits a diverse range of functions and has been identified as an inhibitor of apoptosis [50]. Bim, a pro-apoptotic protein of the Bcl-2 family that is involved in the mitochondrial apoptosis pathway, was shown to be one of the substrates of REGγ. Bim can activate the caspase family and then activate the mitochondrial apoptotic pathway. Caspases also regulate gasdermin family members, inducing the cleavage of gasdermin to produce the active N-terminal fragment, which ultimately leads to pyroptosis [11, 51, 52]. In this study, Bim knockout was found to alleviate pyroptosis caused by the defective expression of REGγ and reduce pyroptosis-induced release of inflammatory factors. Therefore, it plays a regulatory role in sepsis.

Sepsis is a critical condition that results in a high mortality rate [53]. Patients suffer from multiple organ damage and cell pyroptosis, the latter of which is considered one of the reasons for its high mortality rate [54–56]. In this study, a sepsis model was constructed through CLP, and it was found that REGγ was blocked in WT sepsis mice, possibly due to inhibited expression of transcription factors. Next, sepsis was successfully induced in *REGγ*^-/-^ mice, in which heightened organ damage and upregulated expression of inflammatory factors were observed compared to WT mice. Based on these discoveries, it is hypothesized that REGγ has the potential to mitigate sepsis by inhibiting pyroptosis. Moreover, in subsequent investigations using clinical samples, it was found that patients with sepsis had impaired REGγ expression. Nonetheless, a more comprehensive understanding of the mechanism requires further exploration.

**Fig. 7:**
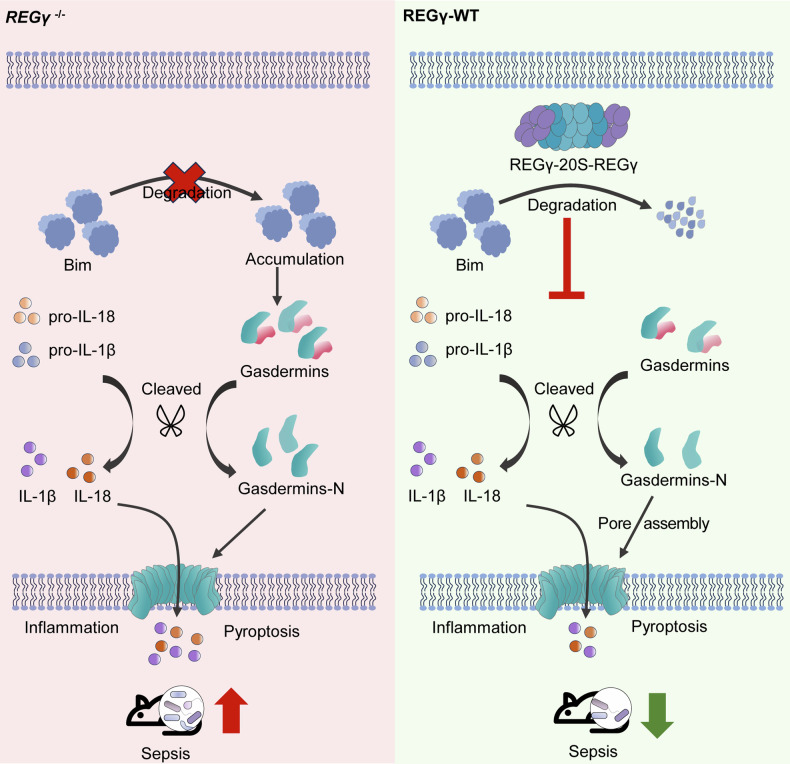
Model of REGγ-Bim-pyroptosis pathway in cells to induce sepsis. Normally, REGγ is able to degrade pro-apoptotic protein Bim through a non-ubiquitin-dependent pathway, thus impeding the cell death process. However, the degradation of Bim is blocked when the expression of REGγ is inhibited, which promotes the accumulation of Bim. Accumulated Bim promotes the continuous activation of the caspase family, followed by the cleavage of gasdermin, and finally leads to pyroptosis. The absence of REGγ promotes excessive pyroptosis of macrophages, ultimately aggravating sepsis.

In summary, this study demonstrated that REGγ regulates pyroptosis by degrading Bim, and its suppression of pyroptosis proved to be advantageous in the context of sepsis. The REGγ mRNA level was downregulated under stimulation with bacterial infection or toxic drugs since the levels of its transcription factors were decreased. The degradation of Bim by the REGγ-20S proteasome was then prevented, resulting in the accumulation of Bim in the body. The high Bim levels upregulate the cleavage of gasdermin, causing enhanced production of the active N-terminus and finally leading to pyroptosis. In sepsis, the downregulation of REGγ promotes macrophage pyroptosis, resulting in inflammation and aggravating sepsis symptoms (Fig. 7). Maintaining normal expression of REGγ can alleviate sepsis symptoms, which suggests that it may be a novel treatment target. Bim, a protein not previously associated with the regulation of pyroptosis and sepsis, has now been revealed to play a role in the control of inflammatory cell death, marking its potential significance in sepsis treatment. This newfound understanding suggests that the REGγ-Bim axis’s regulation of pyroptosis could offer novel treatment strategies for various inflammatory diseases.

## Materials and methods

### Cell lines, antibodies, plasmids, and transfection

HeLa, HEK293T, HK-2, HaCat, and MH-S cell lines were purchased from ATCC authenticated by STR profiling, and tested for mycoplasma contamination. These cells were cultured in Dulbecco’s modified Eagle’s medium (DMEM) with 10% fetal bovine serum (FBS, Sunrise) and 1% penicillin/streptomycin (Invitrogen). HeLa shN cells and HeLa sh*REGγ* cells were previously generated by stable integration of a control vector or a retroviral REGγ-specific shRNA. Bone marrow-derived macrophages (BMDMs) were obtained from 6-10 weeks-old male mice and cultured in minimum Eagle’s medium (MEM) medium (Invitrogen) with 10% FBS, 1% penicillin-streptomycin and 10 ng/mL M-CSF (PeproTech, USA) according to a previous study [57]. Primary peritoneal macrophages (PMs) were obtained from 6–8 weeks-old male mice injected with 3% thioglycolate broth (Sigma) cultured in DMEM [58]. Primary PBMCs were extracted from human blood for western blot analysis [57].

Anti-DFNA5/GSDME-N-terminal (ab215191), anti-GSDMD (ab209845), anti-REGγ (ab157157), anti-Mcl-1(ab32087) and anti-Bcl-2 (ab182858) were purchased from Abcam. Anti-HA tag (51064-2-AP) was purchased from Proteintech. Anti-caspase-1 (T510200) was purchased from Abmart. Anti-Bim (#2933), anti-Bad (#9239), anti-Puma (#4976, #98672), anti-Bid (#2002, #2003), anti-Bak (#12105), anti-Bax (#2772), anti-Bcl-xL (#2764), anti-PARP (#9542), anti-caspase3 (#9662) were all purchased from Cell Signaling Technology. Anti-β-Actin (M177-3), and anti-Flag tag (M185-3L) were purchased from MBL.

Plasmid human pcDNA3.1-Flag-REGγ was previously generated. Plasmids pSG5-HA-BimEL, pSG5-HA-BimL, and pSG5-HA-BimS were constructed based on the sequence of human BimEL, BimL, and BimS gene searched from NCBI. The universal primers for three isoforms of Bim are forward: 5’-CGGAATTCATGGCAAAGCAACCTTCTG-3’, reverse: 5’-GCTCTAGA TCAATGCATTCTCCACACC-3’. As for BimL and BimS, additional primers were required for overlapping PCR. The middle primers for BimL are forward: 5’-ATGGGTGCTGGGCTCCTGTCTTGTGGCTCTGTCTGTAGGG-3’, reverse: 5’-CCCTACAGACAGAGCCACAAGACAGGAGCCCAGCACCCAT-3’. The middle primers for BimS are forward: 5’-TCTCTGGGCGCATATCTGCAGGTTCAGCCTGCCTCATGGA-3’, reverse: 5’-TCCATGAGGCAGGCTGAACCTGCAGATATGCGCCCAGAGA-3’. Lipofectamine™ 3000 (L3000075, Invitrogen) was used for transient transfection of plasmids into 293 T cells according to the recommended procedure in the manufacturer’s instruction. Cells were then harvested after 48 h of transfection for the next detection.

### Assays of cell death and flow cytometry

Cell death was assessed by Hoechst 33342/PI Double stain kit. Cells were seeded in 12-well plates. HeLa cell lines were stimulated with 40 μM cisplatin (P4393, Sigma-Aldrich) for certain hours. BMDMs were pre-priming with 1 μg/mL LPS (L2630, Sigma-Aldrich) for certain hours before being treated with 5 mM ATP (A2383, Sigma-Aldrich) for 1 h. Then cells were stained with Hoechst 33342/PI according to the manufacturer’s instructions. Stained cells are pictured with a fluorescence microscope. Hoechst 33342 was blue fluorescence which can penetrate the intact cell membrane. PI was red fluorescence which can only stain cells with damaged cell membranes. PI uptake was calculated by quantifying the stained cells in five separate fields of each group.

Cell viability was measured by LDH release assay (Beyotime). HeLa cells and BMDMs were seeded in a 96-well plate and treated as previously described. Collect the supernatant and detect LDH release according to the kit instructions. Absorbance was measured at 490 nm to obtain the data. Relative LDH release was calculated on the basis of the background and maximum absorbance in each well and was calculated as LDH release = (sample-background)/ (maximum-background) (%).

For the analysis of lymphocytes in the spleen from cisplatin intraperitoneal injected mice, the samples were stained with APC/Cy7 zombie NRTM for dead cells (Biolegend, CA, USA), PerCP/Cy5.5 anti-mouse CD45 (Biolegend, CA, USA), FITC anti-mouse CD3 (Biolegend, CA, USA) for 20 min on ice in the dark. Flow cytometer analysis was performed at a fixed sample volume, to assess the lymphocyte counts in the spleen from injected mice.

To determine the nature of cell death of cisplatin-treated HeLa cells or LPS-treated BMDMs, samples were stained with Annexin V-APC/PI apoptosis Kit (E-CK-A217, Elabscience) for 15 min at room temperature in the dark. Data were analyzed by FlowJo analytical software (TreeStar) on an LSRFortessa flow cytometer (BD Bioscience, CA, USA).

### Biochemical assay

The serum concentration of alanine aminotransferase (ALT), creatine kinase (CK), and blood urea nitrogen (BUN) in healthy mice or CLP-induced sepsis mice were detected by Servicebio Co., Ltd (Wuhan, China). The whole blood of mice was left to rest overnight at 4 °C and then centrifuged at 3000 rpm for 10 min to obtain serum.

### CRISPR–Cas9 deletion of Bim

Bim knockout cell lines were generated by CRISPR-Cas9 technology. Briefly, two guide RNAs were designed to target Bim: 5’-CACCGGACAATTGCAGCCTGCGGAG-3’ and 5’-AAACCTCCGCAGGCTGCAATTGTCC-3’. They were cloned into the gRNA-expression plasmid lentiCRISPR-V2-Hygromycin. Plasmids were transfected into 293T cells together with the packing plasmids pSPAX2 and pMD2G. Forty-eight hours later, cell supernatants were collected to infect HeLa shN cells and HeLa sh*REGγ* cells for another 24 h. After that, cells were treated with hygromycin at a specific concentration and then cultured in 96-well plates for two weeks to screen Bim-deficient clones. Cells were identified by western blot.

### Western blot, qPCR, and co-immunoprecipitation

Cells or tissues were resuspended in Lysis buffer followed by heat denature for 15 min. Samples were resolved by SDS-PAGE gel and then transferred to the nitrocellulose membrane. After an overnight primary antibody incubation at 4 °C, followed by a fluorescent-labeled secondary antibody, protein bands were visualized by the LI-COR Image Studio software.

qPCR was performed as described. Cells or tissues were lysis with RNAiso Plus (9109, Takara) to extract total RNA according to the manufacturer’s instructions. RNA was then reverse transcribed into cDNA using Superscript II Reverse Transcriptase (R222-01, Vazyme, China). Quantitative PCR was performed with SYBQ mix (Q711-03, Vazyme, China). The mRNA level of target genes was normalized to that of 18S. Primers for qPCR are presented in Table S1.

Plasmids of pcDNA3.1-Flag-REGγ and pSG5-HA-Bim were transfected into 293 T cells. After 48 h expression, cells were harvested and lysed in 0.2% NP-40 isotonic buffer. Specific proteins were immunoprecipitated by anti-Flag beads. The pellet was suspended in a Lysis loading buffer and detected by western blot.

### Histopathological analysis

Organs from mice were collected and fixed in 4% paraformaldehyde (Sangon Biotech, Shanghai, China). The fixed time varies according to the type of organs. Paraffin-embedded tissues were sectioned at a thickness of 5 μm, followed by hematoxylin and eosin (H&E) staining.

### Animal studies

*Bim*^flox/flox^*LysM*^+/+^ mice were purchased from GemPharmatech Co., Ltd by CRISPR-Cas9 technology, and crossed with *LysM*-Cre to generate macrophage-specific *Bim* knockout mice (*Bim*^flox/flox^*LysM* ^Cre^). *REGγ*^*-/-*^ mice were kindly provided by Dr. John J. Monaco at the University of Cincinnati (Barton et al., 2004). Mice were housed in ventilated cages at 22–24 °C in a specific pathogen-free (SPF) animal room.

The CLP-induced sepsis model was constructed as previously described. In brief, 6-10 weeks C57BL/6 male mice were used. Mice were anesthetized and the cecum was exposed under aseptic conditions. Ligate at the medium of the cecum and the single through-and-through puncture was made with a 20 G needle. Extrude a small amount of fecal material from the perforated sites. After that, the peritoneum was closed layer by layer, and injected 1 mL sterile saline by subcutaneous administration. Organs were collected for western blot, histopathological analysis, and wet/dry ratio analysis [59]. Blood was collected for biochemical assay.

To establish a mouse model of chemotherapy drug injury, mice were injected with 10 mg/kg cisplatin by peritoneal and sacrificed at 3–5 days. The small intestine of mice was taken for histopathological analysis and other organs were taken for western blot.

### Human samples

Fresh peripheral blood from patients diagnosed with sepsis based on the third sepsis definition (SEPSIS-3) [12] or from healthy donors were collected. Informed consent was acquired from all enrolled patients. The information on patients is presented in Table S2.

### Statistical analysis

Prism software (GraphPad Software) was used for statistical analyses. The intensity of the western blot results was analyzed by densitometry using ImageJ software. Values were shown as mean ± SD. Within each group, there is an estimate of variation, and the variance between groups is similar. Specific details of statistical analyses conducted were described in the figure legends or main text. Two-tailed Student’s *t-*test was used for comparisons between the two groups. Statistical significance from three or more groups was calculated by one-way or two-way ANOVA with Tukey’s or Sidak’s multiple comparisons test. Survival curves were compared by the Log-rank test. *P* < 0.05 was considered statistically significant. A data point is excluded if it deviates from the mean with more than three standard deviations. Investigators were not blinded to the group allocation during the experiment and when assessing the outcome in all experiments including animal experiments.

### Ethical statement

We have complied with all relevant ethical regulations for animal testing and research. The research protocol was approved by the Animal Experiment Ethics Committee of East China Normal University (Approval number: ARXM2022145). The clinical study was approved by Shanghai Fifth People’s Hospital, Fudan University (Approval number: 2023125×1).

## Supplementary information

Table S1

Table S2

Supplementary figure information

original western blots of all figures

Supplementary movie S1

Supplementary movie S2

## Data Availability

All data needed to evaluate the conclusions in the paper are present in the paper or the Supplementary Materials.
