# Supplementary material for: Ubiquitin-independent degradation of Bim blocks macrophage pyroptosis in sepsis-related tissue injury
Source: Cell Death Dis. 2024 Sep 30;15(9):703. doi: 10.1038/s41419-024-07072-z (PMC11442472; doi:10.1038/s41419-024-07072-z)
Supplement: Supplementary file 3 — Supplementary figure information [file 41419_2024_7072_MOESM3_ESM.pdf]

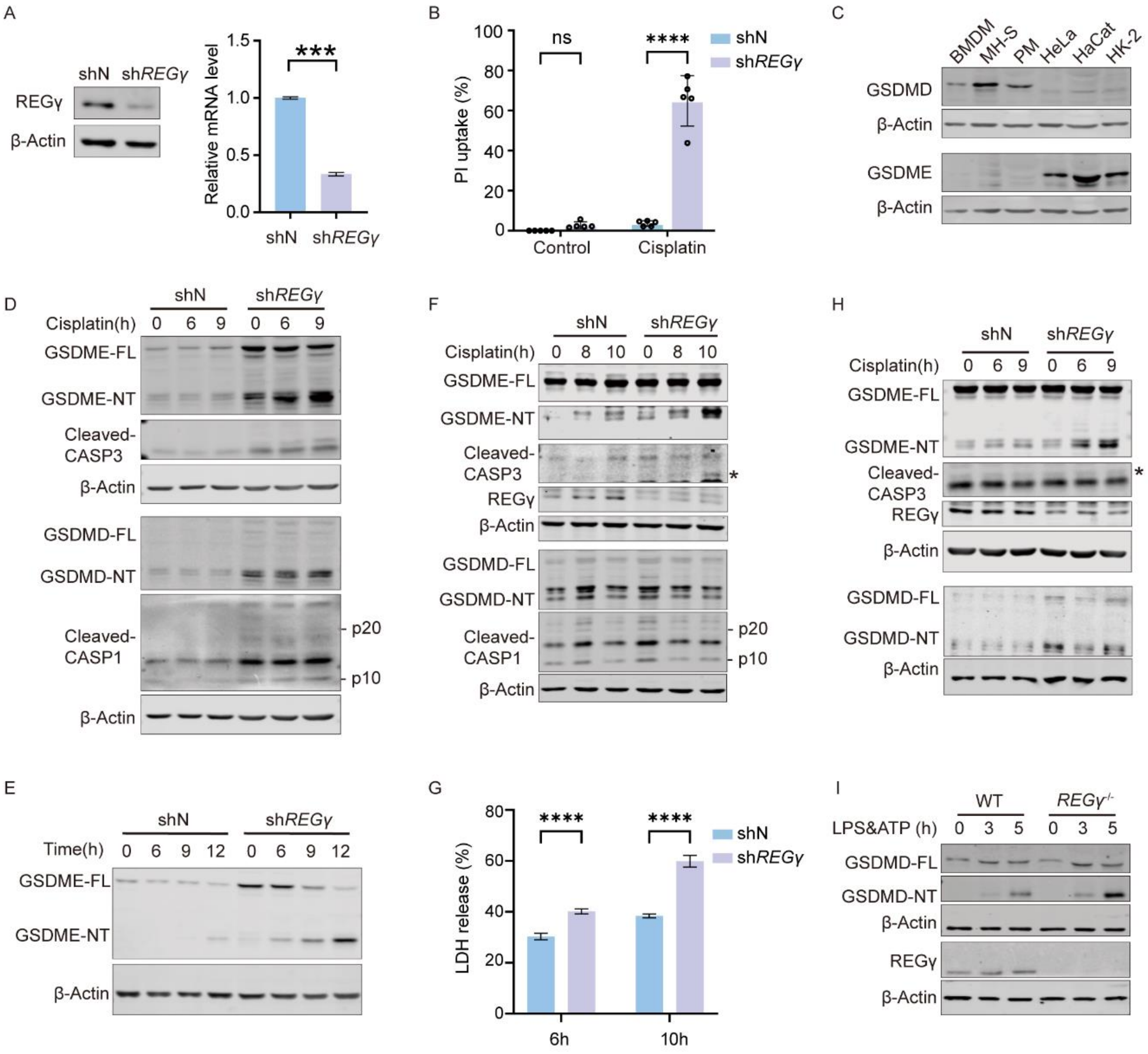

Supplementary Figure 1. REGγ deficiency promoted pyroptotic cell death. (A) Protein level and mRNA level in HeLa shN and HeLa sh*REGγ*. Differences were measured by T test. **(B)** Quantitated results in Fig. 1A. Five fields were randomly selected to count the cells with Hoechst 33342/PI double staining. Differences were measured by two-way ANOVA. (**C)** GSDME and GSDMD expression detected by western blot in different cell lines. **(D)** The expression of GSDME and GSDMD in HeLa cells 40 μM under cisplatin treatment. **(E)** HeLa cells were treated with 40 μM cisplatin at different time and then analyzed for the expression of GSDME by western blot. **(F)** HaCat cells were treated with 40 μM cisplatin at different time and then analyzed for the

expression of GSDME by western blot. The asterisk marked the band being analyzed. **(G)** LDH release into HaCat culture medium under 40 μM cisplatin stimulation was measured. Differences were measured by two-way ANOVA. **(H)** HK-2 cells were treated with 40 μM cisplatin at different time and then analyzed for the expression of GSDME by western blot. The asterisk marked the band being analyzed . **(I)** PMs were treated with 500 ng/mL LPS followed by 5 mM ATP at different time and then analyzed for the expression of GSDMD by western blot. FL: full length, NT: N-terminus. Data are means ± SD taken from three technical replicates. ns, not significant, $*P < 0.05$, $**P < 0.01$, $***P < 0.001$, $****P < 0.0001$.

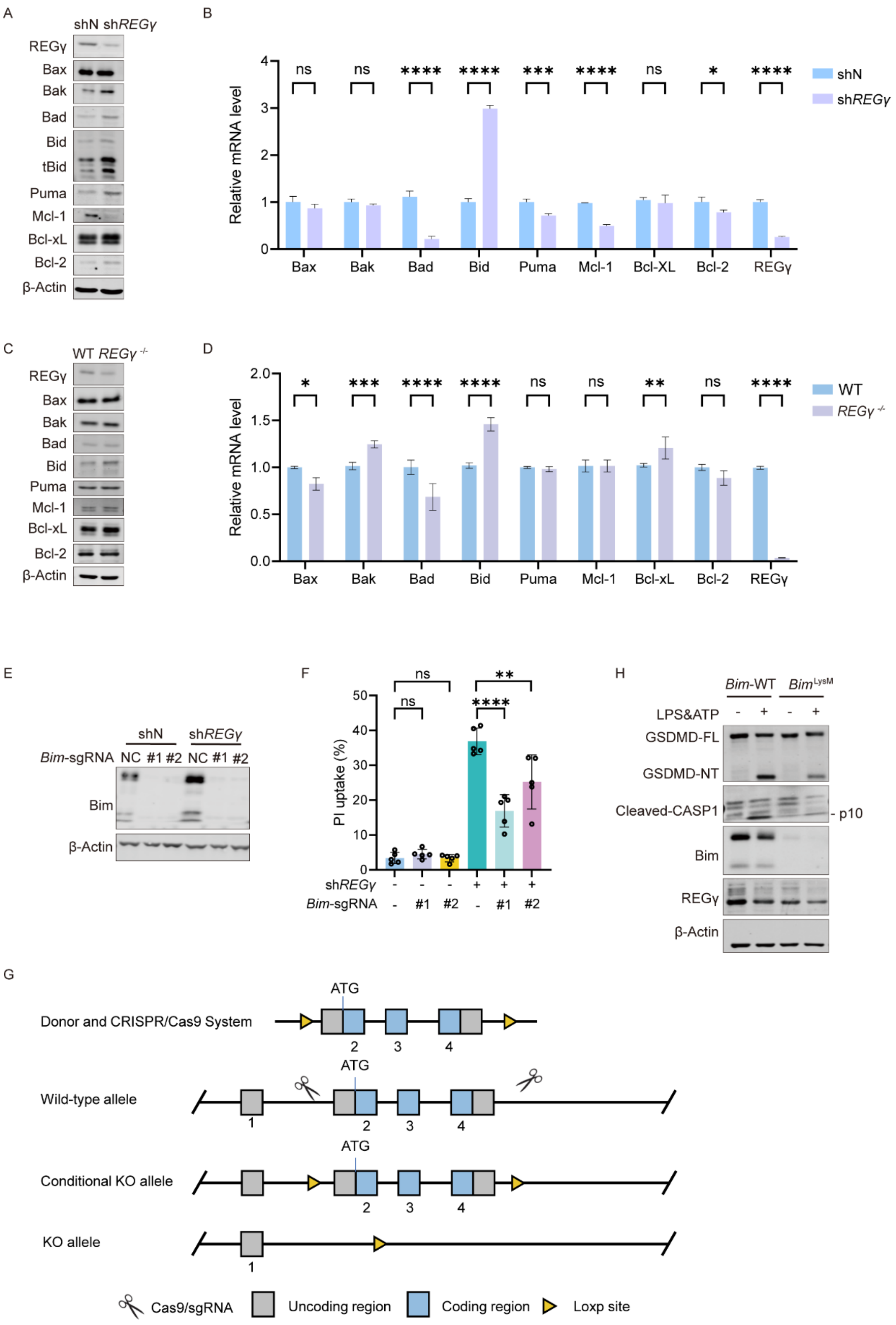
A
shN shREGγ
REGγ
Bax
Bak
Bad
Bid
tBid
Puma
Mcl-1
Bcl-xL
Bcl-2
β-Actin
B
Relative mRNA level
ns ns **** **** *** **** ns * ****
Bax Bak Bad Bid Puma Mcl-1 Bcl-XL Bcl-2 REGγ
shN
shREGγ
C
WT REGγ -/-
REGγ
Bax
Bak
Bad
Bid
Puma
Mcl-1
Bcl-xL
Bcl-2
β-Actin
D
Relative mRNA level
* *** **** **** ns ns ** ns ****
Bax Bak Bad Bid Puma Mcl-1 Bcl-xL Bcl-2 REGγ
WT
REGγ -/-
E
shN shREGγ
Bim-sgRNA NC #1 #2 NC #1 #2
Bim
β-Actin
F
PI uptake (%)
ns ** ns ****
shREGγ - - - + + +
Bim-sgRNA - #1 #2 - #1 #2
H
Bim-WT Bim LysM
LPS&ATP - + - +
GSDMD-FL
GSDMD-NT
Cleaved-CASP1
- p10
Bim
REGγ
β-Actin
G
Donor and CRISPR/Cas9 System
Wild-type allele
Conditional KO allele
KO allele
ATG
Cas9/sgRNA
Uncoding region
Coding region
Loxp site

Supplementary Figure 2. Loss of Bim alleviated the promotive effect of REGγ deficiency on pyroptosis. (A) The protein levels of Bcl-2 family members in HeLa cells. **(B)** The mRNA levels of Bcl-2 family members in HeLa cells. **(C)** The protein levels of Bcl-2 family members in BMDMs. **(D)** The mRNA levels of Bcl-2 family members in BMDMs. **(E)** HeLa *Bim*-knockout cells and HeLa *Bim*-knockout-sh*REGγ* cells were constructed. Cells were analyzed by western blot. NC means cells were transfected with sgRNA negative control. #1/2 means two different *Bim*-specific sgRNAs. **(F)** Quantitated results of Hoechst 33342/PI staining in Figure 2F. **(G)** The schematic diagram to generated *Bim* conditional knockout mice ($Bim^{\text{flox/flox}}$). According to the structure of *Bim* gene, exon2-4 of *Bim* transcript is recommended as the knockout region. The region contains all coding sequence. Knock out the region will result in disruption of protein function. CRISPR/Cas9 technology was used to modify *Bim* gene. The brief process is as follows: gRNA was transcribed in vitro, donor was constructed. Cas9, gRNA and Donor were microinjected into the fertilized eggs of C57BL/6JGpt mice. Fertilized eggs were transplanted to obtain positive F0 mice which were confirmed by PCR and sequencing. A stable F1 generation mouse model was obtained by mating positive F0 generation mice with C57BL/6JGpt mice. **(H)** BMDMs from $Bim^{\text{flox/flox}}LysM^{+/+}$ mice (*Bim*-WT) and macrophage-specific *Bim* knockout $Bim^{\text{flox/flox}}LysM^{\text{Cre}}$ mice (Bim^{LysM}) were treated with 1 μg/mL LPS and 5 mM ATP for 6h and analyzed by western blot. Differences were measured by two-way ANOVA. Data are means ± SD taken from three technical replicates. ns, not significant, *$P < 0.05$, **$P < 0.01$, ***$P < 0.001$.

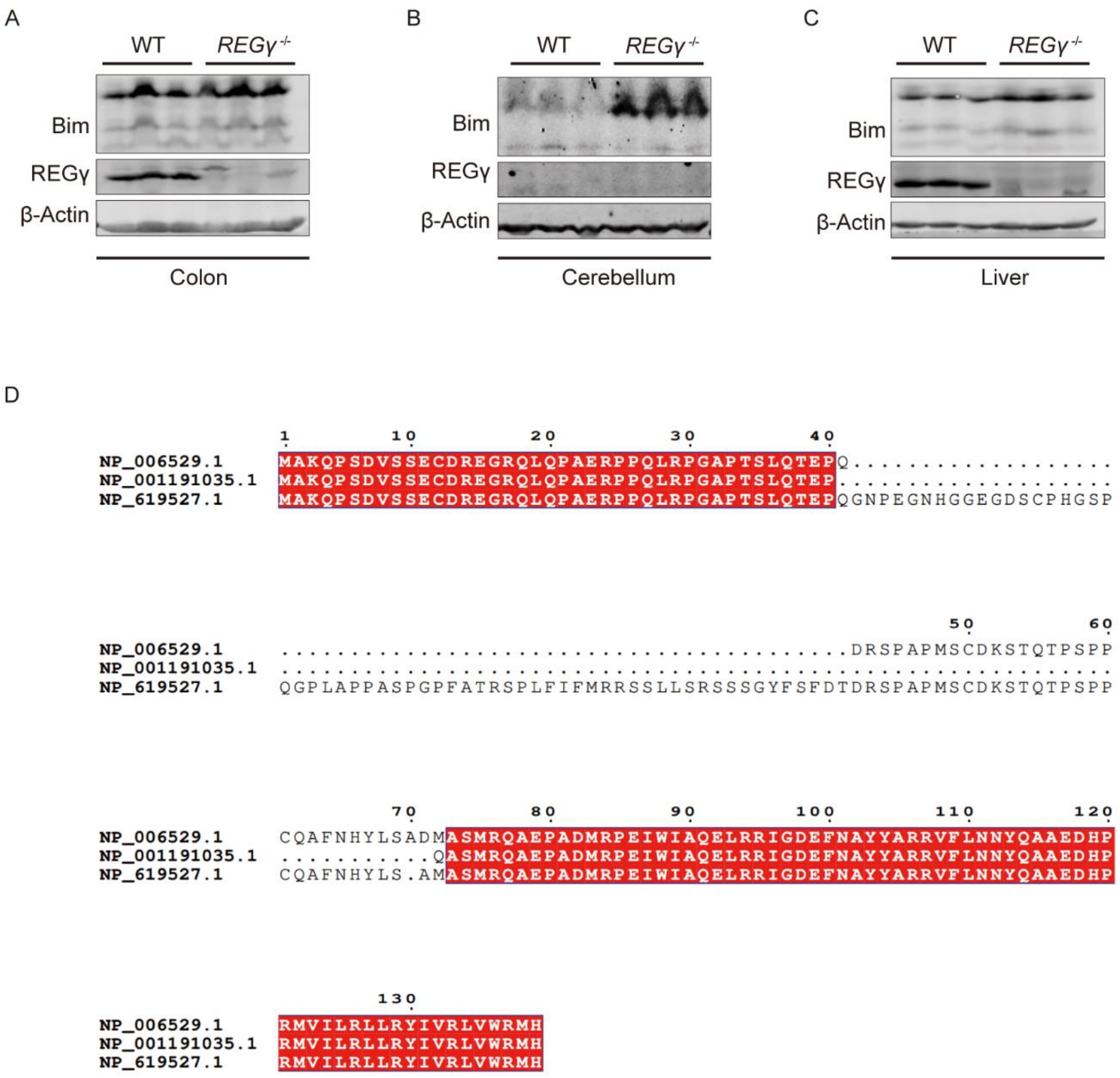

Supplementary Figure 3. REGγ mediated ubiquitin-ATP-independent degradation of Bim. (A-C) Colon, cerebellum and liver from WT mice and *REGγ*$^{-/-}$ mice were assessed for Bim expression by western blot, respectively. **(D)** Sequence alignment of BimEL (down, NP_619527.1), BimL (up, NP_0062529.1) and BimS (medium, NP_001191035.1).

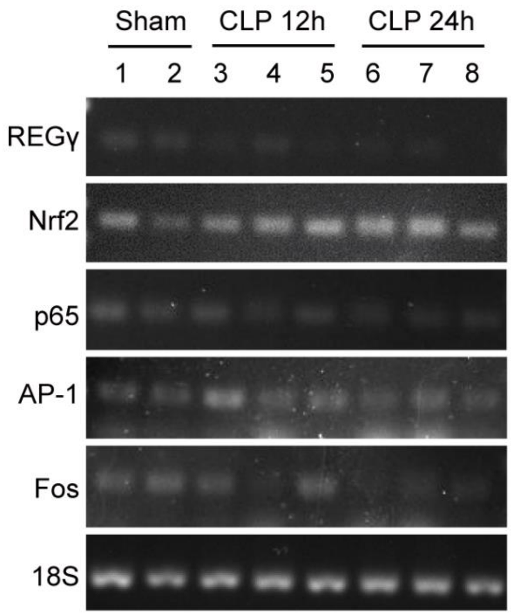

Supplementary Figure 4. REGγ is downregulated in CLP mice possibly due to the downregulation of its transcription factors. The expression level of transcription factors regulating REGγ were measure by DNA gel in BMDMs.

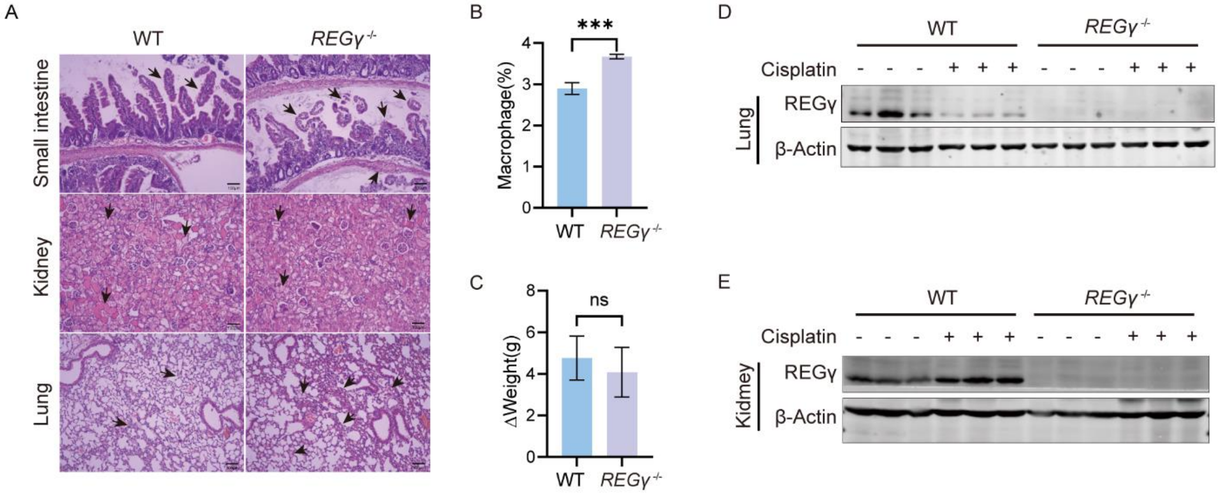

Supplementary Figure 5. Cisplatin injection downregulated the expression of REGγ in the lung tissue. (A) Male 6-10 weeks WT mice and *REGγ*$^{-/-}$ mice were injected with 10 mg/kg cisplatin intraperitoneally. The tissue injury of small intestine, kidney and lung were exhibited by H&E staining. **(B)** The number of macrophages in spleen from WT mice and *REGγ*$^{-/-}$ mice injected with cisplatin. **(C)** The weight loss of WT mice and *REGγ*$^{-/-}$ mice injected with 10 mg/kg cisplatin intraperitoneally from first day to the fifth day. **(D)** The expression of REGγ in the lung from WT mice and *REGγ*$^{-/-}$ mice injected with cisplatin. **(E)** The expression of REGγ in the kidney from WT mice and *REGγ*$^{-/-}$ mice injected with cisplatin. Differences were measured by T test. Data are means ± SD taken from three technical replicates. ns, not significant, *$P < 0.05$, **$P < 0.01$, ***$P < 0.001$.

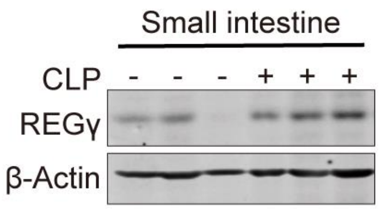

Supplementary Figure 6. The expression of REGγ in small intestine in CLP-induced sepsis mice. The expression of REGγ in small intestine from WT mice with CLP operation.

Movie S1. Cell morphology of HeLa shN cell under cisplatin stimulation with PI staining

Movie S2. Cell morphology of HeLa sh*REGγ* cell under cisplatin stimulation with PI staining
