# Supplementary material for: Ubiquitin-independent degradation of Bim blocks macrophage pyroptosis in sepsis-related tissue injury
Source: Cell Death Dis. 2024 Sep 30;15(9):703. doi: 10.1038/s41419-024-07072-z (PMC11442472; doi:10.1038/s41419-024-07072-z)

Figure 1D

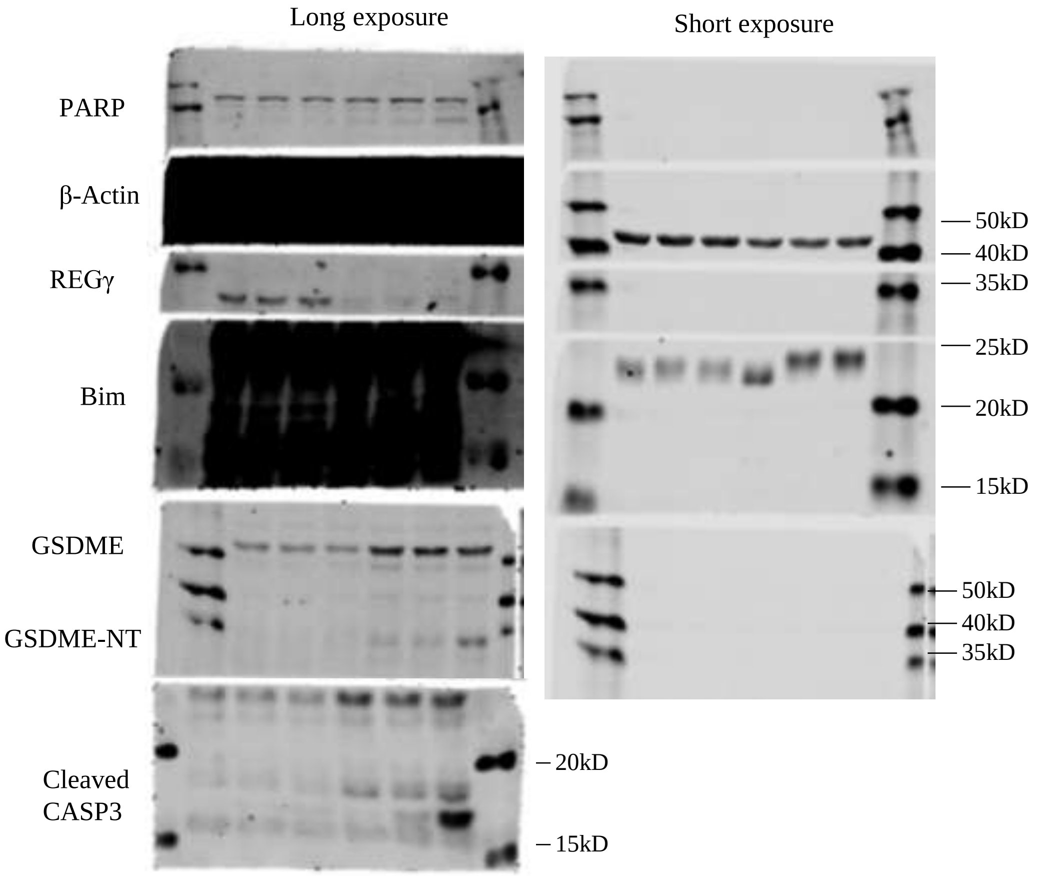

Figure 1H

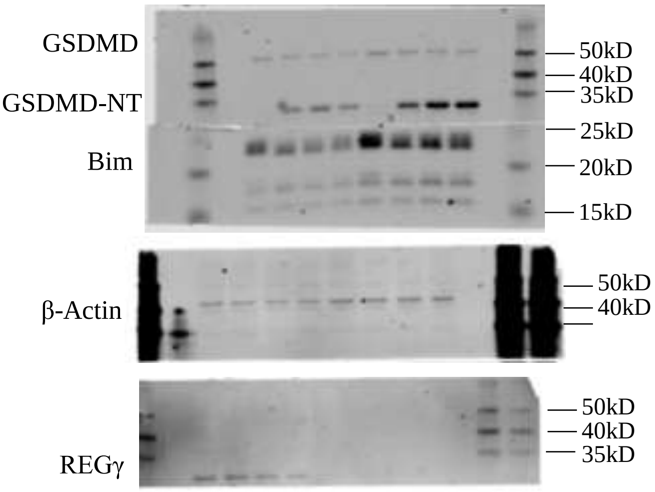

Figure 2A

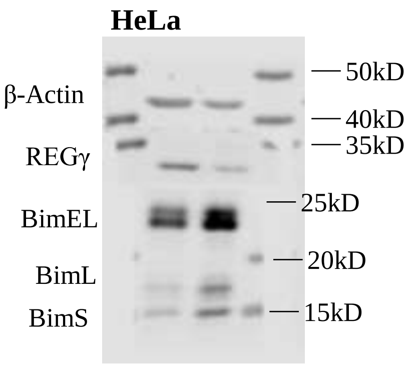

Figure 2B

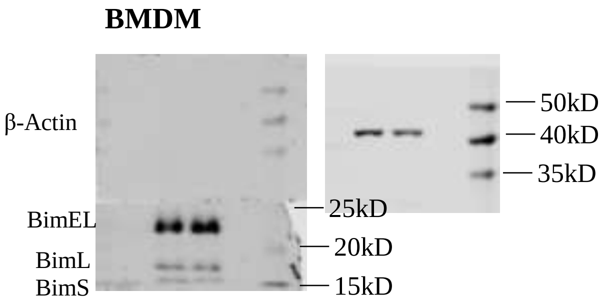

Figure 2G

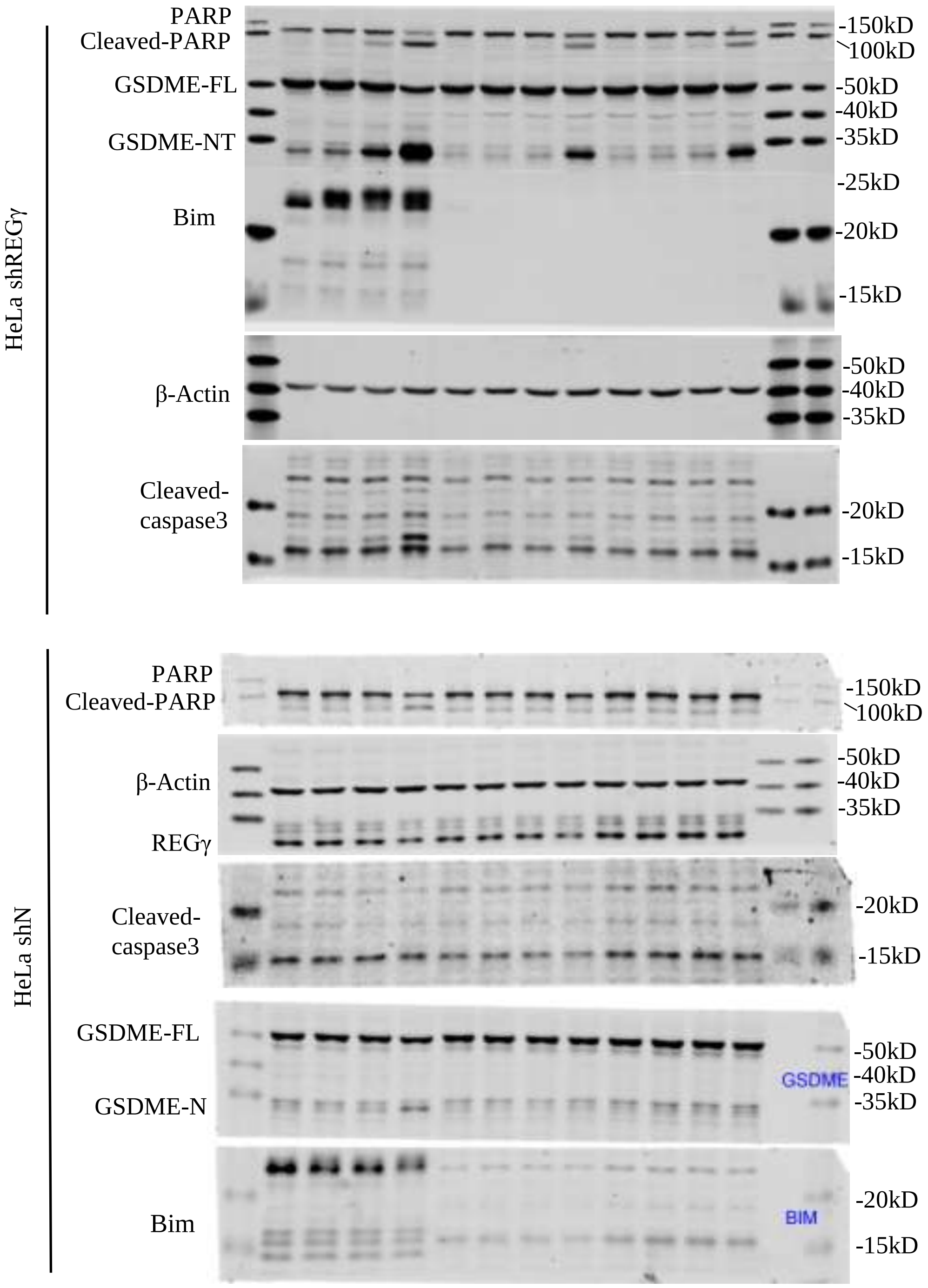

Figure 3B

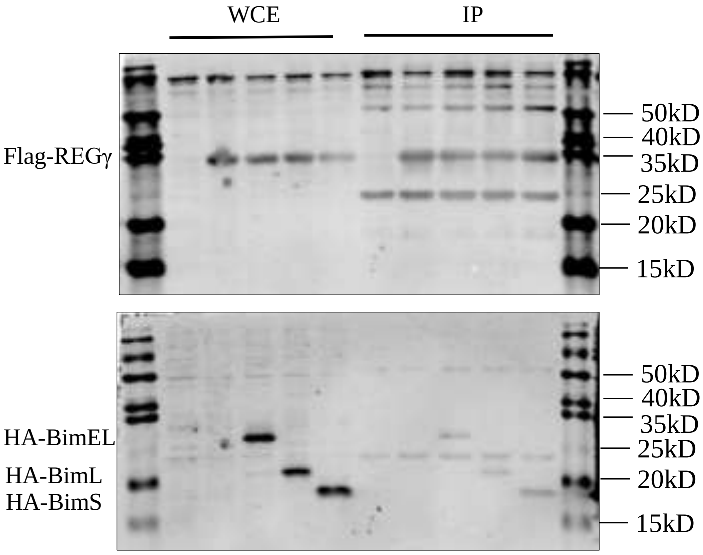

Figure 3C

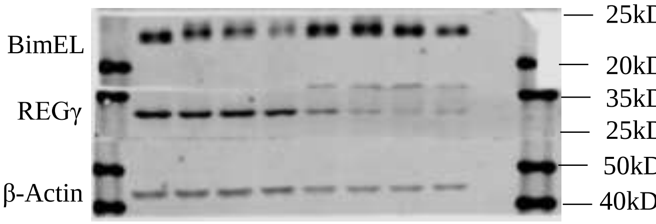

Figure 3D

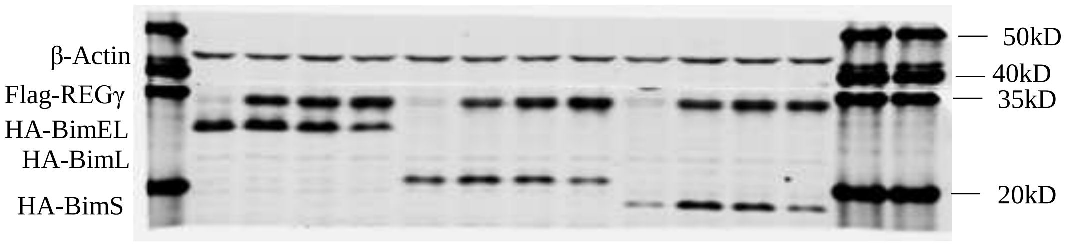

Figure 3E

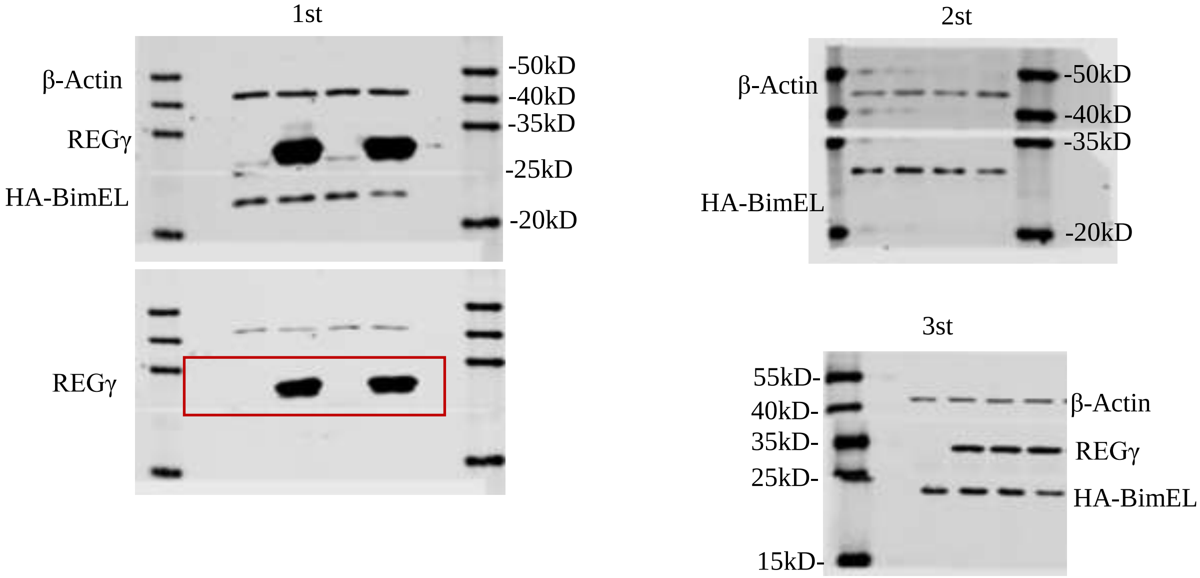

Figure 4G

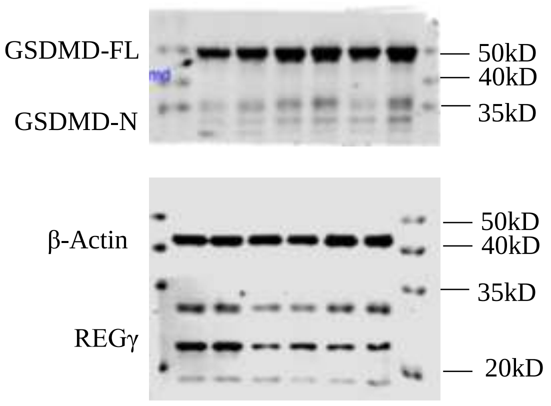
GSDMD-FL
GSDMD-N
50kD
40kD
35kD
β-Actin
REGγ
50kD
40kD
35kD
20kD

Figure 5D

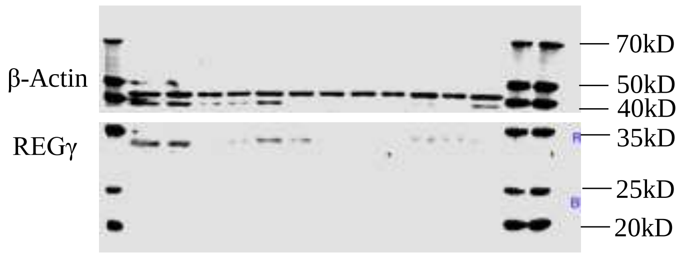

Figure 5E

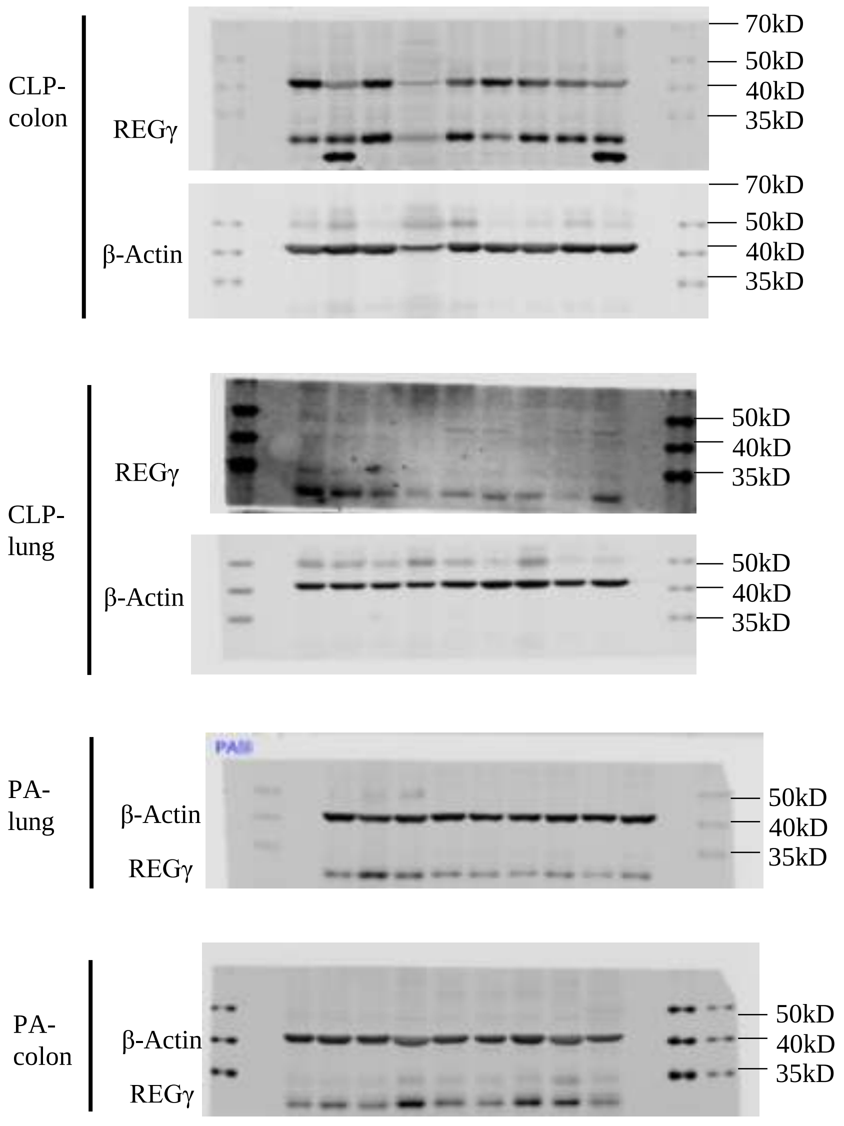

Figure 6G

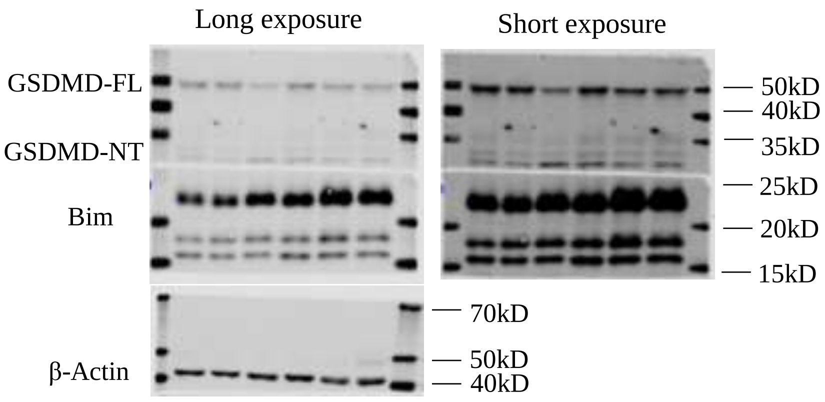
Long exposure
Short exposure
GSDMD-FL
GSDMD-NT
Bim
β-Actin
50kD
40kD
35kD
25kD
20kD
15kD
70kD
50kD
40kD

Fig.S1A

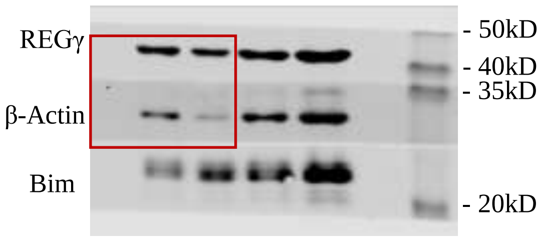

Fig.S1C

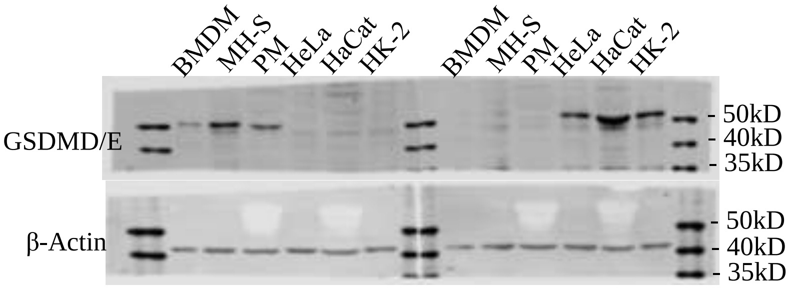

Fig.S1D

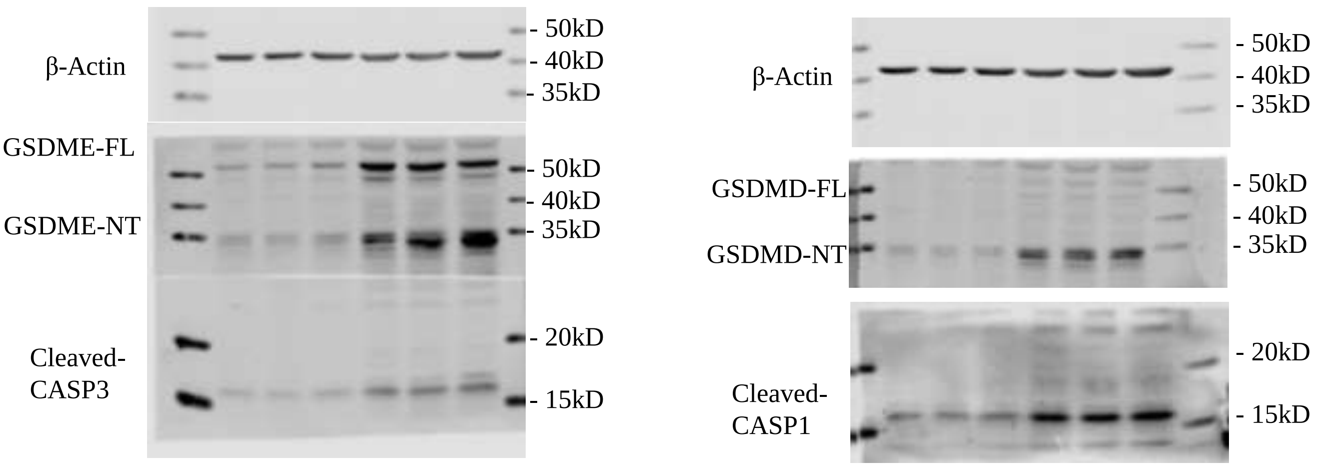

Fig.S1E

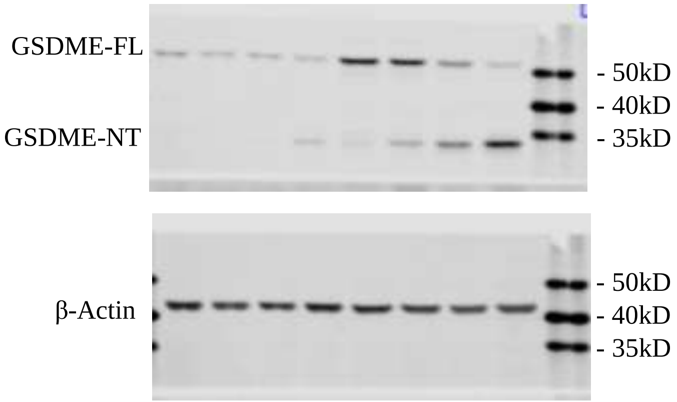

Fig.S1F

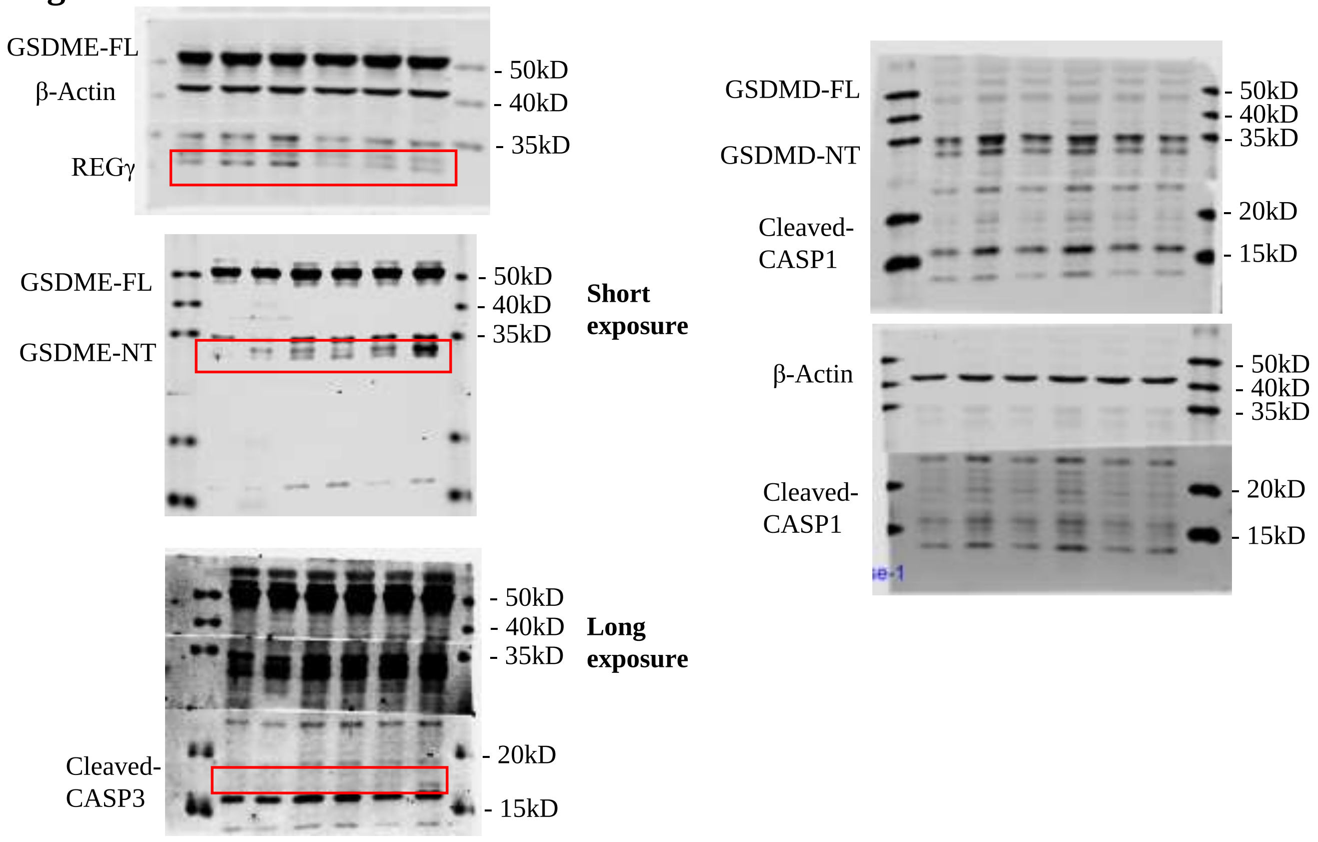

Fig.S1H

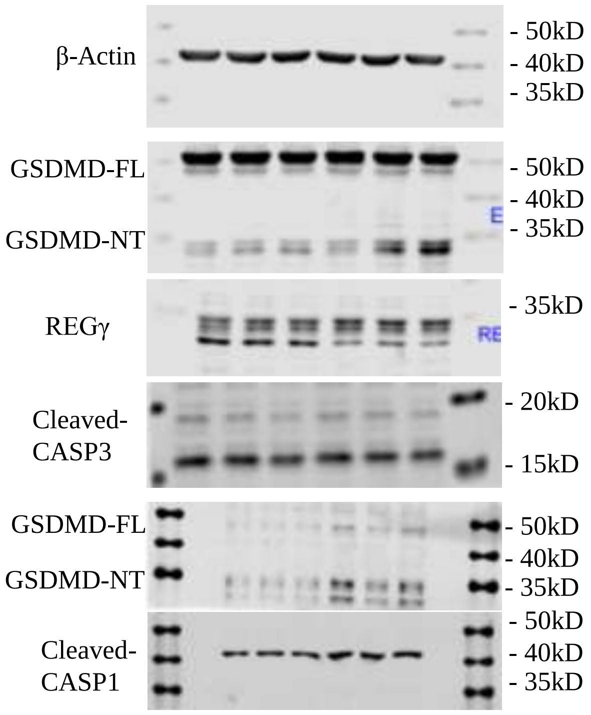

Fig.S1I

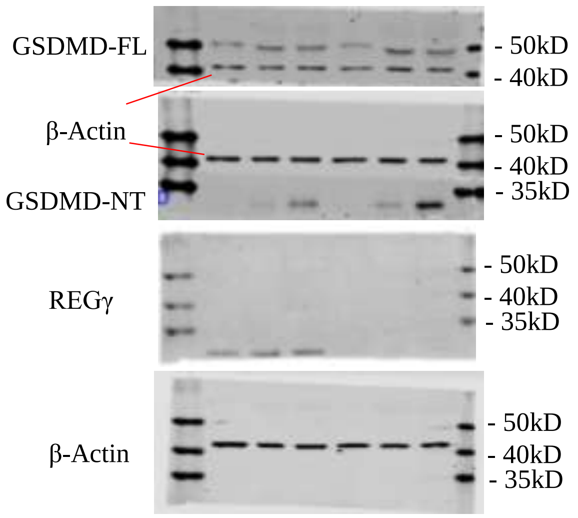

Fig.S2A

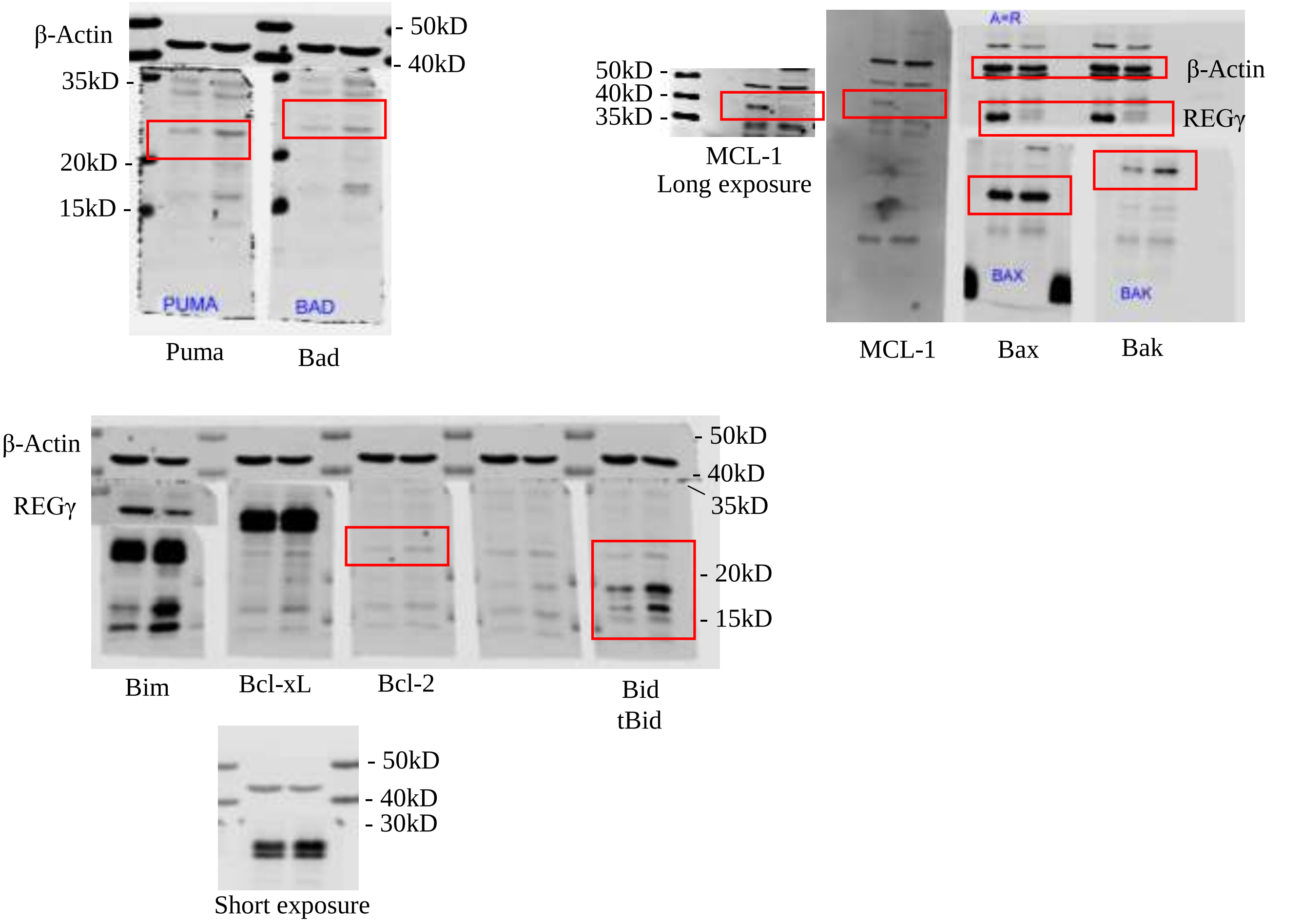

Fig.S2B

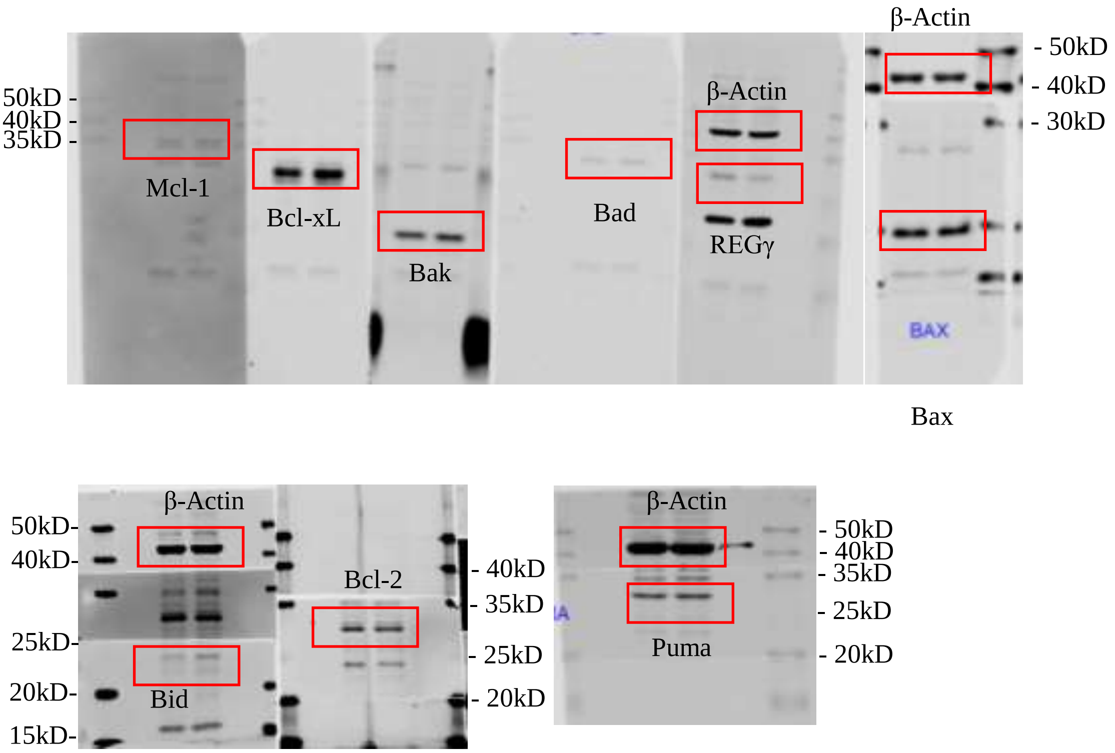

Fig.S2E

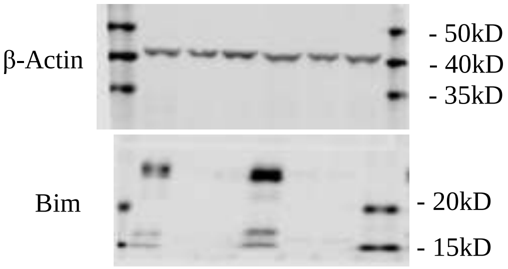

Fig.S2H

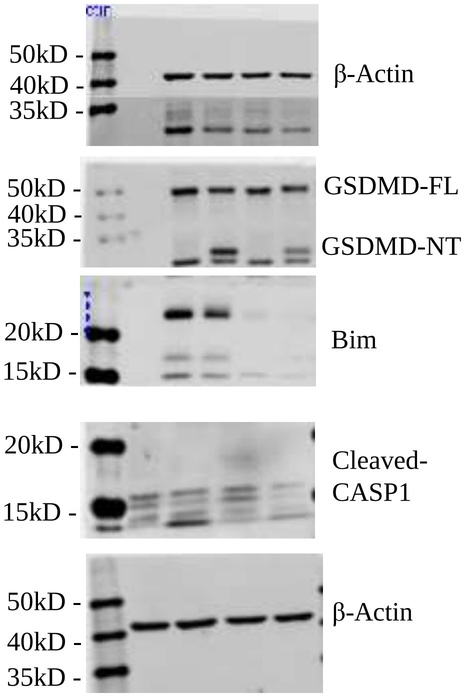

Fig.S5E

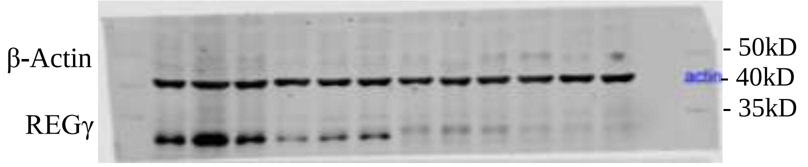

Fig.S5F

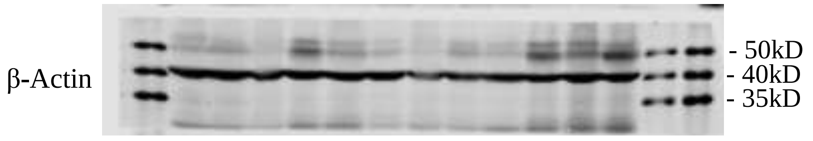

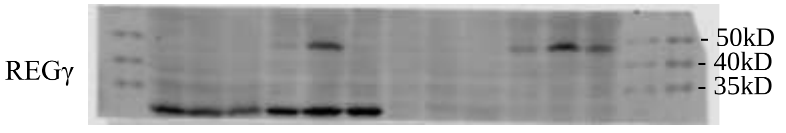

Fig.S6

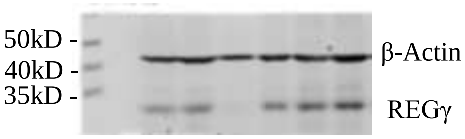

Supplement: Supplementary file 4 — original western blots of all figures [file 41419_2024_7072_MOESM4_ESM.pdf]
